# On the solution stability of parabolic optimal control problems

**DOI:** 10.1007/s10589-023-00473-4

**Published:** 2023-03-20

**Authors:** Alberto Domínguez Corella, Nicolai Jork, Vladimir M. Veliov

**Affiliations:** Institute of Statistics and Mathematical Methods in Economics, Vienna University of Technology, Vienna, Austria

**Keywords:** 49K20, 35K58, 49K40, 49J40

## Abstract

The paper investigates stability properties of solutions of optimal control problems constrained by semilinear parabolic partial differential equations. Hölder or Lipschitz dependence of the optimal solution on perturbations are obtained for problems in which the equation and the objective functional are affine with respect to the control. The perturbations may appear in both the equation and in the objective functional and may nonlinearly depend on the state and control variables. The main results are based on an extension of recently introduced assumptions on the joint growth of the first and second variation of the objective functional. The stability of the optimal solution is obtained as a consequence of a more general result obtained in the paper–the metric subregularity of the mapping associated with the system of first order necessary optimality conditions. This property also enables error estimates for approximation methods. A Lipschitz estimate for the dependence of the optimal control on the Tikhonov regularization parameter is obtained as a by-product.

## Introduction

Let $\Omega \subset {\mathbb {R}}^n$, $1\le n\le 3$, be a bounded domain with Lipschitz boundary $\partial \Omega$. For a finite $T>0$, denote by $Q:=\Omega \times (0,T)$ the space-time cylinder and by $\Sigma :=\partial \Omega \times (0,T)$ its lateral boundary. In the present paper, we investigate the following optimal control problem:

1 $$\begin{aligned} \mathrm{(P)}\ \ \min _{u \in \mathcal {U}}\bigg \{ J(u) := \int _Q L(x,t,y(x,t),u(x,t))\,\textrm{d}x\,\textrm{d}t\bigg \}, \end{aligned}$$

subject to

2 $$\begin{aligned} \left\{ \begin{array}{lll} \frac{\partial y}{\partial t}+\mathcal Ay+f(\cdot ,y)=u\ &{}\text { in }\ Q,\\ y=0 \text { on } \Sigma ,\quad y(\cdot ,0) = y_0\ {} &{}\text { on } \Omega .\\ \end{array} \right. \end{aligned}$$

Here $y: Q \rightarrow {\mathbb {R}}$ is the state, $u: Q \rightarrow {\mathbb {R}}$ is the control and ${\mathcal {A}}$ is an elliptic operator. For functions $u_a, u_b\in L^\infty (Q)$ such that $u_{a} < u_{b}$ a.e in *Q*, the set of feasible controls is given by

3 $$\begin{aligned} \mathcal {U}:= \{u \in L^\infty (Q) \vert \ u_a \le u \le u_b\ \text { for a.a. } (x,t) \in Q\}. \end{aligned}$$

Denote by $y_u$ the unique solution to the semilinear parabolic Eq. (2) that corresponds to control $u\in L^r(Q)$, where *r* is a fixed number satisfying the inequality $r>1+\frac{n}{2}$. The objective integrand in (1) is defined as

4 $$\begin{aligned} L(x,t,y,u):=L_0(x,t,y)+(my+g)u, \end{aligned}$$

where *m* is a number, *g* is a function in $L^\infty (Q)$ and $L_0$ satisfies appropriate smoothness condition (see Assumption 2 in Sect. 1.1).

The goal of the present paper is to obtain stability results for the optimal solution of problem (1)–(3). The meaning of “stability” we focus on, is as follows. Given a reference optimal control ${\bar{u}}$ and the corresponding solution ${\bar{y}}$, the goal is to estimate the distance (call it $\Delta$) from the optimal solutions $(u,y_u)$ of a disturbed version of problem (1)–(3) to the pair $({\bar{u}}, {\bar{y}})$, in terms of the size of the perturbations (call it $\delta$). The perturbations may enter either in the objective integrand or in the state equation, and the meaning of “distance” and “size” in the previous sentence will be clarified in the sequel in terms of appropriate norms. If an estimation $\Delta \le \mathrm{const.} \delta ^\theta$ holds with $\theta \in (0,1)$, we talk about *Hölder stability*, while in the case $\theta = 1$ we have *Lipschitz stability*.

A powerful technique for establishing stability properties of the solutions of optimization problems is based on regularity properties of the system of first order necessary optimality conditions (see e.g. [18]). In the case of problem (1)–(3), these are represented by a *differential variational inequality* (see e.g. [16, 25]), consisting of two parabolic equations (the primal equation (1) and the corresponding adjoint equation) and one variational inequality representing the condition for minimization of the Hamiltonian associated with the problem. The Lipschitz or Hölder stability of the solution of problem (1)–(3) is then a consequence of the property of *metric subregularity* (see [15, 18]) of the mapping defining this differential variational inequality. An advantage of this approach is that it unifies in a compact way the study of stability of optimal solutions under a variety of perturbations (linear or nonlinear). Therefore, the main result in the present paper focuses on conditions for metric subregularity of the mapping associated with the first order optimality conditions for problem (1)–(3). These conditions are related to appropriate second order sufficient optimality conditions, which are revisited and extended in the paper. Several results for stability of the solutions are obtained as a consequence.

The commonly used second order sufficient optimality conditions for ODE or PDE optimal control problems involve a *coercivity condition*, requiring strong positive definiteness of the objective functional as a function of the control in a Hilbert space. We stress that problem (1)–(3) is affine with respect to the control variable and such a coercivity condition is not fulfilled. The theory of sufficient optimality conditions and the regularity theory for affine optimal control of ODE systems have been developed in the past decade, see [24] and the bibliography therein. Sufficient conditions for weak or strong local optimality for optimal control problems with constraints given by elliptic or parabolic equations are developed in [2, 3, 5, 8, 10, 12, 17]. A detailed discussion thereof is provided in Sect. 2.1. In contrast with the elliptic setting, there are only a few stability results for semilinear parabolic optimal control problems. Results in this regard for a tracking type objective functional were obtained for instance in [9, 10] where stability with respect to perturbations in the objective functional was studied, and in [11], where stability with respect to perturbations in the initial data was investigated. We mention that for a linear state equation and a tracking type objective functional, Lipschitz estimates were obtained in [30] under an additional assumption on the structure of the optimal control. More comprehensive discussion about the sufficiency theory and stability can be found in Sect. 2.

The main novelty in the present paper is the study of the subregularity property of the optimality mapping associated with problem (1)–(3). In contrast with the case of coercive problems, our assumptions in the affine case jointly involve the first and the second order variations of the objective functional with respect to the control. These assumptions are weaker than the ones in the existing literature in the context of sufficient optimality conditions, however, they are strong enough to imply metric subregularity of the optimality mapping. The subregularity result is used to obtain new Hölder- and Lipschitz estimates for the solution of the considered optimal control problem. An error estimate for the Tikhonov regularization is obtained as a consequence.

The obtained subregularity result provides a base for convergence and error analysis for discretization methods applied to problem (1)–(3). The point is, that numerical solutions of the discretized versions of the problem typically satisfy approximately first order optimality conditions for the discretized problem and after appropriate embedding in the continuous setting (1)–(3), satisfy the optimality conditions for the latter problem with a residual depending on the approximation and the discretization error. Then the subregularity property of the optimality mapping associated with (1)–(3) provides an error estimate. Notice that the (Lipschitz) stability of the solution alone is not enough for such a conclusion, and this is an important motivation for studying subregularity of the optimality mapping rather than only stability of the solutions. However, we do not go into this subject, postponing it to a later paper based on the present one.

The paper is organized as follows. The analysis of the optimal control problem (1)–(3) begins in Sect. 2. We recall the state of the art regarding second order sufficient conditions for weak and strong (local) optimality, as well as known sufficient conditions for stability of optimal controls and states under perturbations. In Sect. 3 we formulate and discuss the assumptions on which our further analysis on sufficiency and stability is based. The strong subregularity of the optimality mapping is proved in Sect. 4. In Sect. 5, we obtain stability results for the optimal control problem under non-linear perturbations, postponing some technicalities to Appendix A. Finally, we support the theoretical results with some examples.

### Preliminaries

We begin with some basic notations and definitions. Given a non-empty, bounded and Lebesgue measurable set $$X\subset {\mathbb {R}}^n$$, we denote by $$L^p(X)$$, $$1\le p\le \infty $$, the Banach spaces of all measurable functions $$f: X \rightarrow {\mathbb {R}}$$ for which the usual norm $$\Vert f\Vert _{L^p(X)}$$ is finite. For a bounded Lipschitz domain $$X\subset {\mathbb {R}}^n$$ (that is, a set with Lipschitz boundary), the Sobolev space $$H^1_0(X)$$ consists of functions that vanish on the boundary (in the trace sense) and that have weak first order derivatives in $$L^2(X)$$. The space $$H_0^1(X)$$ is equipped with its usual norm denoted by $$\Vert \cdot \Vert _{H^1_0(X)}$$. By $$ H^{-1}(X)$$ we denote the topological dual of $$H_0^1(X)$$, equipped with the standard norm $$\Vert \cdot \Vert _{H^{-1}(X)}$$. Given a real Banach space *Z*, the space $$L^p(0,T\text {; } Z)$$ consist of all strongly measurable functions $$y:[0,T]\rightarrow Z$$ that satisfy

$$\begin{aligned} \Vert y\Vert _{L^p(0,T\text {; }Z)}:=\Big (\int _0^T \Vert y(t) \Vert _{Z}^p\,\textrm{d}t\Big )^{\frac{1}{p}}<\infty \qquad \text{ if } \; 1\le p<\infty , \end{aligned}$$

or, for $$p=\infty $$,

$$\begin{aligned} \Vert y\Vert _{L^\infty (0,T\text {; }Z)}:=\text {inf}\{M\in \mathbb {R}\ \vert \ \Vert y(t)\Vert _{Z}\le M \text { for a.e } t\in (0,T)\}<\infty . \end{aligned}$$

The Hilbert space *W*(0, *T*) consists of all of functions in $L^2(0,T\text {; }H^1_0(\Omega ))$ that have a distributional derivative in $L^2(0,T\text {; }H^{-1}(\Omega ))$, i.e.

$$\begin{aligned} W(0,T):=\Bigg \{ y\in L^2(0,T;H^1_0(\Omega ))\Big \vert \ \frac{\partial y}{\partial t}\in L^2(0,T;H^{-1}(\Omega )) \Bigg \}, \end{aligned}$$

which is endowed with the norm

$$\begin{aligned} \Vert y\Vert _{W(0,T)}:=\Vert y\Vert _{L^2(0,T;H^1_0(\Omega ))}+\Vert \partial y/\partial t \Vert _{L^2(0,T;H^{-1}(\Omega ))}. \end{aligned}$$

The Banach space $C([0,T]\text {; }L^2(\Omega ))$ consists of all continuous functions $y:[0,T]\rightarrow L^2(\Omega )$ and is equipped with the norm $\max _{t\in [0,T]}\Vert y(t)\Vert _{L^2(\Omega )}$. It is well known that *W*(0, *T*) is continuously embedded in $C([0,T]\text {; }L^2(\Omega ))$ and compactly embedded in $L^2(Q)$. The duality pairing between a Banach space *X* and its dual is denoted by $\langle \cdot , \cdot \rangle _X$. For proofs and further details regarding spaces involving time, see [14, 20, 27, 31].

The following assumptions, close to those in [3, 5, 6, 8, 10–13], are standing in all the paper, together with the inequality

5 $$\begin{aligned} r> \max \Big \{2,1+\frac{n}{2} \Big \} \end{aligned}$$

for the real number *r* that appears in some assumptions and many statements below (we also remind that $n \in \{1,2,3\}$). Although for $n=1$ it is admissible to have $r=2$ (instead of $r > 2$), we keep the above restriction in order to treat all the cases in a unified way.

#### Assumption 1

The operator ${\mathcal {A}}:H^1_0(\Omega )\rightarrow H^{-1}(\Omega )$, is given by

$$\begin{aligned} {\mathcal {A}} y=-\sum _{i,j=1}^{n}\partial _{x_j}(a_{i,j}(x)\partial _{x_i}y ), \end{aligned}$$

where $a_{i,j}\in L^{\infty }(\Omega )$ satisfy the uniform ellipticity condition

$$\begin{aligned} \exists \lambda _{\mathcal {A}}>0:\ \lambda _{\mathcal {A}}\vert \xi \vert ^2\le \sum _{i,j=1}^{n} a_{i,j}(x)\xi _{i}\xi _{j} \;\; \text { for all } \xi \in \mathbb {R}^n \;\; \text {and a.a.}\ x\in \Omega . \end{aligned}$$

The functions $f, \, L_0: Q \times {\mathbb {R}}\longrightarrow \mathbb {R}$ of the variables (*x*, *t*, *y*), and the “initial” function $y_0$ have the following properties.

#### Assumption 2

For every $y \in {\mathbb {R}}$, the functions $f(\cdot ,\cdot ,y) \in L^{r}(Q)$, $L_0(\cdot ,\cdot ,y) \in L^{1}(Q)$, and $y_0 \in L^{\infty }(\Omega )$. For a.e. $(x,t) \in Q$ the first and the second derivatives of *f* and $L_0$ with respect to *y* exist and are locally bounded and locally Lipschitz continuous, uniformly with respect to $(x,t) \in Q$. Moreover, $\frac{\partial f}{\partial y}(x,t,y) \ge 0$ for a.e. $(x,t) \in Q$ and for all $y\in {\mathbb {R}}$.

#### Remark 1

The last condition in Assumption 2 can be relaxed in the following way:

$$\begin{aligned} \exists C_{f}\in {\mathbb {R}}:\ \frac{\partial f}{\partial y}(x,t,y)\ge C_f \ \text { a.a. }(x,t)\in Q \text { and }\forall y\in {\mathbb {R}}, \end{aligned}$$

see [5, 8]. However, this leads to complications in the proofs.

### Facts regarding the linear and the semilinear equation

Let $0\le \alpha \in L^{\infty }(Q)$ and $u\in L^2(Q)$. We consider solutions of the following linear variational equality for $h \in W(0,T)$ with $h(\cdot ,0)=0$:

6 $$\begin{aligned} \int _{0}^{T}\big \langle \frac{\partial h}{\partial t}+{\mathcal {A}} h,\psi \big \rangle _{H^1_0(\Omega )}\,dt =\int _{0}^{T}\langle u-\alpha h,\psi \rangle _{L^2(\Omega )}\,dt \end{aligned}$$

for all $\psi \in L^2(0,T, H^1_0(\Omega ))$, that is, for weak solutions of the Eq. (2) with $f(x,t,h):=\alpha (x,t) h$ and zero initial datum.

#### Theorem 1

Let $0\le \alpha \in L^\infty (Q)$ be given.

1. For each $u\in L^2(Q)$ the linear parabolic equation (6) has a unique weak solution $h_{u}\in W(0,T)$. Moreover, there exists a constant $C_2 >0$ independent of *u* and $\alpha$ such that
  7 $$\begin{aligned} \Vert h_u\Vert _{L^2(0,T,H^1_0(\Omega ))}\le C_2 \Vert u\Vert _{L^{2}(Q)}. \end{aligned}$$
2. If, additionally, $u\in L^{r}(Q)$ (we remind (5)) then the weak solution $h_u$ of (6) belongs to $W(0,T)\cap C(\bar{Q})$. Moreover, there exists a constant $C_r>0$ independent of *u* and $\alpha$ such that
  8 $$\begin{aligned} \Vert h_u\Vert _{L^2(0,T,H^1_0(\Omega ))}+\Vert h_u\Vert _{C({\bar{Q}})}\le C_r \Vert u\Vert _{L^{r}(Q)}. \end{aligned}$$

Besides the independence of the constants $C_2,$ and $C_r$ on $\alpha$ all claims of the theorem are well known, see [29, Theorem 3.13, Theorem 5.5]. A proof of a similar independence statement can be found in [3] for a linear elliptic PDE of non-monotone type. We further remark that item 2 of Theorem 1 is true in dimension $n=1$ even for $r=2$, see [21, Section III.7].

#### Proof

For convenience of the reader, we prove that the estimates are independent of $\alpha$. This is done along the lines of the proof of [3, Lemma 2.2]. By $h_{0,u}$ we denote a solution of (6) for $\alpha =0$. It is well known that in this case there exist positive constants $C_r, C_2$ such that

$$\begin{aligned} \Vert h_{0,u}\Vert _{C({\bar{Q}})}\le C_r \Vert u \Vert _{L^r(Q)},\ \ \Vert h_{0,u} \Vert _{L^2(Q)}\le C_2 \Vert u\Vert _{L^2(Q)}. \end{aligned}$$

To apply this, we decompose *u* in positive and negative parts, $u=u^+-u^-$, $u^+, u^-\ge 0$. By the weak maximum principle [14, Theorem 11.9], it follows that $h_{\alpha ,u^+}, h_{\alpha ,u^-}\ge 0$. Again by the weak maximum principle, the equation

$$\begin{aligned} \frac{\partial }{\partial t}(h_{\alpha ,u^+}-h_{0,u^+})+{\mathcal {A}}(h_{\alpha ,u^+}-h_{0,u^+}) +\alpha (h_{\alpha ,u^+}-h_{0,u^+}) =-\alpha h_{0,u^+} \end{aligned}$$

implies $0\le h_{\alpha ,u^+}\le h_{0,u^+},$ thus $\Vert h_{\alpha ,u^+}\Vert _{C({\bar{Q}})} \le \Vert h_{0,u^+}\Vert _{C({\bar{Q}})}$. By the same reasoning, it follows that $0\le h_{\alpha ,u^-}\le h_{0,u^-}$ and $\Vert h_{\alpha ,u^-}\Vert _{C({\bar{Q}})} \le \Vert h_{0,u^-}\Vert _{C({\bar{Q}})}$. Hence,

$$\begin{aligned} \begin{aligned} \Vert h_{\alpha ,u}\Vert _{C({\bar{Q}})}&\le \Vert h_{\alpha ,u^+}\Vert _{C({\bar{Q}})} + \Vert h_{\alpha ,u^-}\Vert _{C({\bar{Q}})}\le \Vert h_{0,u^+}\Vert _{C({\bar{Q}})} + \Vert h_{0,u^-}\Vert _{C({\bar{Q}})}\\&\le C_r(\Vert u^+ \Vert _{L^r(Q)} +\Vert u^-\Vert _{L^r(Q)} )\le 2C_r \Vert u\Vert _{L^r(Q)}. \end{aligned} \end{aligned}$$

The estimate for $L^2(0,T,H^1_0(\Omega ))$ can be obtained by similar arguments as in [3]. $\square$

The next lemma is motivated by an analogous result for linear elliptic equations [3, Lemma 2.3], although, according to the nature of the parabolic setting, the interval of feasible numbers *s*, is smaller.

#### Lemma 2

Let $u\in L^{r}(Q)$ and $0\le \alpha \in L^\infty (Q)$. Let $h_u$ be the unique solution of (6) and let $p_u$ be a solution of the problem

9 $$\begin{aligned} \left\{ \begin{array}{l} -\frac{\partial p}{\partial t}+{\mathcal {A}}^*p+\alpha p = u\ \text { in }\ Q,\\ p=0 \text { on } \Sigma ,\ p(\cdot ,T)=0\ \text { on } \Omega . \end{array} \right. \end{aligned}$$

Then, for any $s_n\in [1,\frac{n+2}{n})$ there exists a constant $C_{s'_{n}}>0$ independent of *u* and $\alpha$ such that

10 $$\begin{aligned} \max \left\{ \Vert h_u\Vert _{L^{s_n}(Q)},\Vert p_u\Vert _{L^{s_n}(Q)}\right\} \le C_{s'_{n}}\Vert u\Vert _{L^{1}(Q)}. \end{aligned}$$

Here $s'_n$ denotes the Hölder conjugate of $s_n$.

#### Proof

First we observe that by Theorem 1, $h_u \in C({\bar{Q}})\cap W(0,T)$ and as a consequence, $\vert h_u\vert ^{s_n - 1}\text {sign}(h_u) \in L^{s'_n}(Q)$. Moreover, $s_n < \frac{n+2}{n}$ implies that $s'_n >1+ \frac{n}{2}$. By change of variables, see for instance [29, Lemma 3.17], a solution of Eq. (9) transforms into a solution of (6). Thus according to Theorem 1, the solution *q* of

$$\begin{aligned} \left\{ \begin{array}{l} -\frac{\partial q}{\partial t}+{\mathcal {A}}^*q+\alpha q= \vert h_u\vert ^{s_n - 1}\text {sign}(h_u)\ \text { in }\ Q, \\ q=0 \text { on } \Sigma ,\ q(\cdot ,T)=0\ \text { on } \Omega . \end{array} \right. \end{aligned}$$

belongs to $W(0,T)\cap C({\bar{Q}})$ and satisfies

$$\begin{aligned} \Vert q\Vert _{C({\bar{Q}})} \le C_{s'_n}\Vert \vert h_u\vert ^{s_n - 1}\text {sign}(h_u)\Vert _{L^{s'_n}(Q)} = C_{s'_n}\Vert h_u\Vert ^{s_n - 1}_{L^{s_n}(Q)}, \end{aligned}$$

where $C_{s'_n}$ is independent of $\alpha$ and *v*. Using these facts we derive the equalities

$$\begin{aligned} \Vert h_u\Vert ^{s_n}_{L^{s_n}(Q)}&= \int _{Q}\vert h_u\vert ^{s_n}\,\textrm{d}x=\big \langle -\frac{\partial q}{\partial t}+ {\mathcal {A}}^*q+\alpha q,h_u \big \rangle = \big \langle \frac{\partial h_u}{\partial t} +\mathcal Ah_u+\alpha h_u, q \big \rangle \\&= \int _{Q}uq\,\textrm{d}x\le \Vert u\Vert _{L^1(Q)}\Vert q\Vert _{C({\bar{Q}})} \le C_{s'_n}\Vert u\Vert _{L^1(Q)}\Vert h_u\Vert ^{s_n - 1}_{L^{s_n}(Q)}. \end{aligned}$$

This proves (10) for $h_u$. To obtain (10) for $p_u$, one tests (9) with a weak solution of

$$\begin{aligned} \left\{ \begin{array}{l} \frac{\partial h}{\partial t}+\mathcal Ah+\alpha h= \vert q_u\vert ^{s_n - 1}\text {sign}(q_u)\ \text { in }\ Q, \\ h=0 \text { on } \Sigma ,\ h(\cdot ,0)=0\ \text { on } \Omega , \end{array} \right. \end{aligned}$$

and argues in an analogous way. $\square$

Below we remind several results for the semilinear equation (2), which will be used further. The first part of the proof of the next theorem can be found in [4, Theorem 2.1], the second in [5, Theorem 2.1].

#### Theorem 3

For every $u\in L^q(0, T; L^p(\Omega ))$ with $\frac{1}{q}+\frac{n}{2p}<1$ and $q, p \ge 2$ there exists a unique solution $y_u \in L^\infty (Q) \cap W (0, T)$ of (2). Moreover, the following estimates hold

11 $$\begin{aligned}{} & {} \Vert y_u \Vert _{L^\infty (Q)} \le \eta (\Vert u\Vert _{L^q (0,T;L^p (\Omega ))} + \Vert f(\cdot , \cdot , 0)\Vert _{L^q (0,T;L^p (\Omega )) }\nonumber \\{} & {} \quad + \Vert y_0\Vert _{L^\infty (\Omega )} ), \end{aligned}$$

12 $$\begin{aligned}{} & {} \Vert y_u \Vert _{C([0,T];L^2(\Omega ))} + \Vert y_u\Vert _{L^2(0,T;H^1_0(\Omega ))}\le K (\Vert u\Vert _{L^2(Q) }\nonumber \\{} & {} \quad + \Vert f(\cdot , \cdot , 0)\Vert _{L^2(Q)} + \Vert y_0\Vert _{L^2(\Omega )} ), \end{aligned}$$

for a monotone non-decreasing function $\eta : [0, \infty )\rightarrow [0, \infty )$ and some constant *K* both independent of *u*. Finally, if $u_k\rightharpoonup u$ weakly in $L^{q}(0,T;L^p(Q))$, then

13 $$\begin{aligned} \Vert y_{u_k}-y_u\Vert _{L^{\infty }(Q)}+\Vert y_{u_k}-y_u\Vert _{L^2(0,T;H^1_0(\Omega ))}\rightarrow 0. \end{aligned}$$

The differentiability of the control-to-state operator under the assumptions 1 and 2 is well known, see among others [8, Theorem 2.4].

#### Theorem 4

The control-to-state operator ${\mathcal {G}}: L^r(Q)\rightarrow W(0,T)\cap L^{\infty }(Q)$, defined as ${\mathcal {G}}(v):=y_v$, is of class $C^2$ and for every $u,v,w\in L^r(Q)$, it holds that $z_{u,v}:={\mathcal {G}}'(u)v$ is the solution of

14 $$\begin{aligned} \left\{ \begin{array}{l} \frac{d z}{dt}+\mathcal Az+f_y(x,t,y_u) z =v\ \text { in }\ Q, \\ z=0 \ \text { on }\ \Sigma ,\ z(\cdot ,0)=0\ \text { on } \Omega \end{array} \right. \end{aligned}$$

and $\omega _{u,(v,w)}:={\mathcal {G}}''(u)(v,w)$ is the solution of

15 $$\begin{aligned} \left\{ \begin{array}{l} \frac{d z}{dt}+\mathcal Az+f_y(x,t,y_u) z = -f_{yy}(x,t,y_u) z_{u,v } z_{u,w} \ \text { in }\ Q, \\ z=0 \ \text { on }\ \Sigma ,\ z(\cdot ,0)=0\ \text { on } \Omega . \end{array} \right. \end{aligned}$$

In the case $v=w$, we will just write $\omega _{u,v}$ instead of $\omega _{u,(v,v)}$.

#### Remark 2

By the boundedness of ${\mathcal {U}}$ in $L^\infty (Q)$ and by Theorem 3, there exists a constant $M_{\mathcal {U}} > 0$ such that

16 $$\begin{aligned} \max \left\{ \Vert u\Vert _{L^\infty (Q)},\Vert y_u\Vert _{L^\infty (Q)}\right\} \le M_{{\mathcal {U}}} \quad \forall u\in {\mathcal {U}}. \end{aligned}$$

### Estimates associated with differentiability

We employ results of the last subsection to derive estimates for the state Eq. (2) and its linearisation (14). These estimates constitute a key ingredient to derive stability results in the later sections. The next lemma extends [3, Lemma 2.7] from elliptic equations to parabolic ones.

#### Lemma 5

The following statements are fulfilled.

(i) There exists a positive constant $M_2$ such that for every $u,{\bar{u}} \in \mathcal {U}\text { and }v\in L^r(Q)$
  17 $$\begin{aligned} \Vert z_{u,v} - z_{{\bar{u}},v}\Vert _{L^2(Q)}\le M_2\Vert y_u - y_{{\bar{u}}}\Vert _{L^\infty (Q)}\Vert z_{{\bar{u}},v}\Vert _{L^2(Q)}. \end{aligned}$$
(ii) Let $X=L^\infty ( Q)$ or $X=L^2(Q)$. Then there exists $\varepsilon > 0$ such that for every $u, {\bar{u}} \in \mathcal {U}$ with $\Vert y_u - y_{{\bar{u}}}\Vert _{L^\infty (Q)} < \varepsilon$ the following inequalities are satisfied
  18 $$\begin{aligned}&\Vert y_u - y_{{\bar{u}}}\Vert _X \le 2\Vert z_{{\bar{u}},u - {\bar{u}}}\Vert _X \le 3\Vert y_u - y_{{\bar{u}}}\Vert _X, \end{aligned}$$
  19 $$\begin{aligned}&\Vert z_{{\bar{u}},v}\Vert _X \le 2 \Vert z_{u,v}\Vert _X \le 3\Vert z_{{\bar{u}},v}\Vert _X. \end{aligned}$$

The proof is a consequence of Lemma 29 given in Appendix A.

## The control problem

The optimal control problem (1)-(3) is well posed under assumptions 1 and 2. Using the direct method of calculus of variations one can easily prove that there exists at least one global minimizer, see [29, Theorem 5.7]. On the other hand, the semilinear state equation makes the optimal control problem nonconvex, therefore we allow global minimizers as well as local ones. In the literature, weak and strong local minimizers are considered.

### Definition 1

We say that ${\bar{u}} \in {\mathcal {U}}$ is an $L^r (Q)$-weak local minimum of problem (1)-(3), if there exists some $\varepsilon >0$ such that

$$\begin{aligned} J( {\bar{u}}) \le J(u) \ \ \ \forall u \in {\mathcal {U}} \text { with } \Vert u-{\bar{u}}\Vert _{L^r(Q)}\le \varepsilon . \end{aligned}$$

We say that ${\bar{u}} \in {\mathcal {U}}$ a strong local minimum of (P) if there exists $\varepsilon >0$ such that

$$\begin{aligned} J( {\bar{u}}) \le J(u) \ \ \ \forall u \in {\mathcal {U}} \text { with } \Vert y_u-y_{{\bar{u}}}\Vert _{L^{\infty }(Q)}\le \varepsilon . \end{aligned}$$

We say that ${\bar{u}} \in {\mathcal {U}}$ is a strict (weak or strong) local minimum if the above inequalities are strict for $u\ne {\bar{u}}$.

Relations between these types of optimality are obtained in [5, Lemma 2.8].

As a consequence of Theorem 4 and the chain rule, we obtain the differentiability of the objective functional with respect to the control.

### Theorem 6

The functional $J:L^r(Q) \longrightarrow \mathbb {R}$ is of class $C^2$. Moreover, given $u, v, v_1, v_2 \in L^r(Q)$ we have

20 $$\begin{aligned} J'(u)v&=\int _Q\Big (\frac{dL_0}{dy}(x,t,y_u)+mu\Big )z_{u,v}+(my_u+g)v\,\textrm{d}x\,\textrm{d}t\end{aligned}$$

21 $$\begin{aligned}&= \int _Q(p_u+my_u+g)v\,\textrm{d}x\,\textrm{d}t, \end{aligned}$$

22 $$\begin{aligned} J''(u)(v_1,v_2)&= \int _Q\Big [\frac{\partial ^2L}{\partial y^2}(x,t,y_u,u) - p_u\frac{\partial ^2f}{\partial y^2}(x,t,y_u)\Big ] z_{u,v_1}z_{u,v_2}\,\textrm{d}x\,\textrm{d}t \end{aligned}$$

23 $$\begin{aligned}&\quad + \int _Qm(z_{u,v_1}v_2+z_{u,v_2}v_1)\,\textrm{d}x\,\textrm{d}t, \end{aligned}$$

Here, $p_u \in W(0,T) \cap C({\bar{Q}})$ is the unique solution of the adjoint equation

24 $$\begin{aligned} \left\{ \begin{array}{l}\displaystyle -\frac{d p}{dt}+\mathcal {A}^*p + \frac{\partial f}{\partial y}(x,t,y_u)p= \frac{\partial L}{\partial y}(x,t,y_u,u) \text { in } Q,\\ p = 0\text { on } \Sigma , \ p(\cdot ,T)=0\text { on } \Omega .\end{array}\right. \end{aligned}$$

We introduce the Hamiltonian $Q\times {\mathbb {R}}\times {\mathbb {R}}\times {\mathbb {R}} \ni (x,t,y,p,u) \mapsto H(x,t,y,p,u) \in {\mathbb {R}}$ in the usual way:

$$\begin{aligned} H(x,t,y,p,u):=L(x,t,y,u)+p(u-f(x,t,y)). \end{aligned}$$

The local form of the Pontryagin type necessary optimality conditions for problem (1)-(3) in the next theorem is well known (see e.g. [5, 8, 29]).

### Theorem 7

If ${\bar{u}}$ is a weak local minimizer for problem (1)-(3), then there exist unique elements ${\bar{y}}, {\bar{p}}\in W(0,T)\cap L^\infty (Q)$ such that

25 $$\begin{aligned}&\left\{ \begin{array}{l} \frac{d {\bar{y}}}{dt}+\mathcal {A}{\bar{y}} + f(x,t,{\bar{y}}) = {\bar{u}} \text { in } Q,\\ {\bar{y}} = 0\text { on } \Sigma , \ {\bar{y}}(\cdot ,0)=y_0\text { on } \Omega .\end{array}\right. \end{aligned}$$

26 $$\begin{aligned}&\left\{ \begin{array}{l}\displaystyle \frac{d {\bar{p}}}{dt}+\mathcal {A}^*{\bar{p}} = \frac{\partial H}{\partial y}(x,t,{\bar{y}},{\bar{p}},{\bar{u}}) \text { in } Q,\\ {\bar{p}} = 0\text { on } \Sigma , \ {\bar{p}}(\cdot ,T)=0\text { on } \Omega .\end{array}\right. \end{aligned}$$

27 $$\begin{aligned}&\int _Q\frac{\partial H}{\partial u}(x,t,{\bar{y}},{\bar{p}},{\bar{u}})(u - {\bar{u}})\,\textrm{d}x\,\textrm{d}t\ge 0 \quad \forall u \in \mathcal {U}. \end{aligned}$$

### Sufficient conditions for optimality and stability

In this subsection we discuss the state of the art in the theory of sufficient second order optimality conditions in PDE optimal control, as well as related stability results for the optimal solution. For this purpose, we recall the definitions of several cones that are useful in the study of sufficient conditions. Given a triplet $({\bar{y}}, {\bar{p}}, {\bar{u}})$ satisfying the optimality system in Theorem 7, and abbreviating $\frac{\partial {\bar{H}}}{\partial u}(x,t):= \frac{\partial H}{\partial u}(x,t,{\bar{y}},{\bar{p}},{\bar{u}})$, we have from (27) that almost everywhere in *Q*

$$\begin{aligned} {\bar{u}}=u_a\; \text { if }\; \frac{\partial {\bar{H}}}{\partial u}> 0\ \ \ \text { and } \ \ {\bar{u}}= u_b \; \text { if } \; \frac{\partial {\bar{H}}}{\partial u}< 0. \end{aligned}$$

This motivates to consider the following set

28 $$\begin{aligned} \Big \{v\in L^2(Q)\Big \vert v\ge 0\text { a.e. on } [{\bar{u}} =u_a]\text { and } v\le 0 \text { a.e. on } [{\bar{u}} =u_b]\Big \}. \end{aligned}$$

Sufficient second order conditions for (local) optimality based on (28) are given in [5, 8, 10]. Following the usual approach in mathematical programming, one can define the critical cone at ${\bar{u}}$ as follows:

$$\begin{aligned} C_{{\bar{u}}}:=\Big \{v\in L^2(Q)\Big \vert v\text { satisfies }(28)\text { and } v(x,t)= 0\text { if }\Big \vert \frac{\partial {\bar{H}}}{\partial u}(x,t)\Big \vert >0\Big \}. \end{aligned}$$

Obviously, this cone is trivial if $\frac{\partial {\bar{H}}}{\partial u}(x,t) \not = 0$ for a.e. (*x*, *t*) (which implies bang-bang structure of ${\bar{u}}$) thus no additional information can be gained based on $C_{{\bar{u}}}$. To address this issue, it was proposed in [19, 22] to consider larger cones on which second order conditions can be posed. Namely, for $\tau >0$ one defines

29 $$\begin{aligned} D^{\tau }_{{\bar{u}}}&:= \Big \{v\in L^2(Q)\Big \vert v\text { satisfies }(28)\text { and } v(x,t)= 0\text { if }\Big \vert \frac{\partial {\bar{H}}}{\partial u}(x,t)\Big \vert >\tau \Big \}, \end{aligned}$$

30 $$\begin{aligned} G^{\tau }_{{\bar{u}}}&:= \Big \{v\in L^2(Q)\Big \vert v\text { satisfies }(28)\text { and } J'({\bar{u}})(v)\le \tau \Vert z_{{\bar{u}},v} \Vert _{L^1(Q)}\Big \}, \end{aligned}$$

31 $$\begin{aligned} E^{\tau }_{{\bar{u}}}&:= \Big \{v\in L^2(Q)\Big \vert v\text { satisfies }(28)\text { and } J'({\bar{u}})(v)\le \tau \Vert z_{{\bar{u}},v} \Vert _{L^2(Q)}\Big \}, \end{aligned}$$

32 $$\begin{aligned} C^\tau _{{\bar{u}}}&:=D^{\tau }_{{\bar{u}}}\cap G^{\tau }_{{\bar{u}}}. \end{aligned}$$

The cones $D^\tau _{{\bar{u}}}$, $E^{\tau }_{{\bar{u}}} \text { and } G^{\tau }_{{\bar{u}}}$ were introduced in [2, 10] as extensions of the usual critical cone. It was proven in [2, 9, 10] that the condition:

33 $$\begin{aligned} \exists \delta>0, \tau >0 \ \ \text{ such } \text{ that } \ \ J''({\bar{u}})v^2\ge \delta \Vert z_{{\bar{u}},v} \Vert _{L^2(Q)}^2 \ \ \forall v \in G \end{aligned}$$

is sufficient for weak (in the case $G=D^{\tau }_{{\bar{u}}}$) or strong (in the case $G=E^{\tau }_{{\bar{u}}}$) local optimality in the elliptic and parabolic setting. Most recently, the cone $C^\tau _{{\bar{u}}}$ was defined in [5] and also used in [6]. It was proved in [5], that (33) with $G=C^\tau _{{\bar{u}}}$ is sufficient for strong local optimality.

Under (33) it is possible to obtain some stability results. In [10] and [9] the authors obtain Lipschitz stability in the ($L^2-L^\infty$)-sense for the states^1^, under perturbations appearing in a tracking type objective functional and under the assumption that the perturbations are Lipschitz. Further they obtain Hölder stability for the states under a Tikhonov type perturbation. Hölder stability under (33) with exponent 1/2 was proved in [11] with respect to perturbations in the initial condition.

To improve the stability results an additional assumption is needed. This role is usually played by the structural assumption on the adjoint state or more general on the derivative of the Hamiltonian with respect to the control. In the case of an elliptic state equation, [26] uses the structural assumption

34 $$\begin{aligned} \exists \kappa> 0 \text { such that } \ \ \Big \vert \Big \{x \in \Omega : \Big \vert \frac{\partial {\bar{H}}}{\partial u} \Big \vert \le \varepsilon \Big \}\Big \vert \le \kappa \varepsilon \quad \forall \varepsilon > 0. \end{aligned}$$

In the parabolic case this assumption (with $\Omega$ replaced with *Q*) is used in [11]. We recall that the assumption (34) implies that ${\bar{u}}$ is of bang-bang type. Further, (34) implies the existence of a positive constant ${{\tilde{\kappa }}}$ such that the following growth property holds:

35 $$\begin{aligned} J'({\bar{u}})(u-{\bar{u}})\ge {\tilde{\kappa }} \Vert u-{\bar{u}}\Vert _{L^1(\Omega )}^2 \ \forall u\in {\mathcal {U}}. \end{aligned}$$

For a proof see [1, 23] or [28]. If the control constraints satisfy $u_a<u_b$ almost everywhere on $\Omega$, both conditions, (34) and (35) are equivalent, see [17, Proposition 6.4]. In [26], using (34) and (33) with $G=D^\tau _{{\bar{u}}}$, the authors prove $L^1-L^2$-Lipschitz stability of the controls for an elliptic semilinear optimal control problem under perturbations appearing simultaneously in the objective functional and the state equation. Assuming (34), condition (33) may also be weakened to the case of negative curvature,

36 $$\begin{aligned} \exists \delta < {\tilde{\kappa }}, \ \exists \tau >0 \ \ \text{ such } \text{ that } \ \ J''({\bar{u}})v^2\ge -\delta \Vert v \Vert _{L^1(\Omega )}^2 \ \ \forall v\in C^{\tau }_{{\bar{u}}}. \end{aligned}$$

This was done in [12, 13] where it was proved that (34) together with (36) implies, for the semililnear elliptic case, weak local optimality. Lipschitz stability results were also obtained in [17] in the elliptic case. Finally, for a semilinear parabolic equation with perturbed initial data, [11, Theorem 4.6] obtains, under (33) and (34), $L^2-L^2$ and $L^1-L^2$-Hölder stability (see Footnote 1), with exponent 2/3, for the optimal states and controls respectively. Additionally, $L^1-L^\infty$ Lipschitz dependence on perturbations is obtained.

## A unified sufficiency condition

In this section, we introduce an assumption that unifies the first and second order conditions presented in the previous section.

### Assumption 3

Let ${\bar{u}} \in {\mathcal {U}}$. For a number $k\in \{0,1,2\}$, at least one of the following conditions is fulfilled:

($A_k$): There exist constants $\alpha _k,\gamma _k>0$ such that

37 $$\begin{aligned} J'({\bar{u}})(u - {\bar{u}}) + J''({\bar{u}})(u - {\bar{u}})^2 \ge \gamma _k\Vert z_{{\bar{u}},u - {\bar{u}}}\Vert ^{k}_{L^2(Q)}\Vert u -{\bar{u}}\Vert ^{2-k}_{L^1(Q)} \end{aligned}$$

for all $$u \in \mathcal {U}\text { with } \Vert y_u - y_{{\bar{u}}}\Vert _{L^\infty (Q)} < \alpha _k$$.

($B_k$): There exist constants ${\tilde{\alpha }}_k, {\tilde{\gamma }}_k>0$ such that (37) holds for all $u \in \mathcal {U}$ such that $\Vert u - {\bar{u}}\Vert _{L^1(Q)} < {\tilde{\alpha }}_k$.

In the context of optimal control of PDE’s the conditions ($A_0$) and ($B_0$) were first introduced in [17] and for $k=1,2$ in [3]. Condition($B_0$) originates from optimal control theory of ODE’s where it was first introduced in [24] to deal with nonlinear affine optimal control problems. The cases $k=1,2$ are extensions, adapted to the nature of the PDE setting, while the case $k=0$ can be hard to verify if a structural assumption like (34) is not imposed. The conditions corresponding to $k=1,2$ are applicable for the case of optimal controls that need not be bang-bang, especially the case $k=2$ seems natural for obtaining state stability. Condition ($A_k$) implies strong (local) optimality, while condition ($B_k$) leads to weak (local) optimality. As seen below, in some cases the two conditions are equivalent.

For an optimal control problem subject to an semilinear elliptic equation the claim of the next proposition with $k=0$ was proven in [3, Proposition 5.2].

### Proposition 8

For any $k\in \{0,1,2\}$, condition ($A_k$) implies ($B_k$). If ${\bar{u}}$ is bang-bang (that is, ${\bar{u}}(x,t) \in \{ u_a(x,t), u_b(x,t) \}$ for a.e. $(x,t) \in Q$) then conditions ($A_k$) and ($B_k$) are equivalent.

The proof is given in Appendix A.

### Remark 3

We compare the items in Assumption 3 to the ones using (34) and (36) or (33).

1. Condition ($A_0$) is implied by the structural assumption (34) and also allows for negative curvature, similar to (36). For details see [17, Theorem 6.3].
2. Let $g=0$. Condition ($A_1$) is implied by the structural assumption (34) together with (33), that is, by the conditions assumed in [11]. For the convenience of the reader, this is proven in Proposition 16.
3. Let $m,g=0$. Condition ($A_2$) is implied by (33) together with the first order necessary optimality condition. This is a conequence of Corollary 15.

### Sufficiency for optimality of the unified condition

In this subsection we show that conditions ($A_k$) and ($B_k$) are sufficient either for strict weak or strict strong local optimality, correspondingly.

#### Theorem 9

The following holds.

1. Let $m=0$ in (4). Let ${\bar{u}} \in \mathcal {U}$ satisfy the optimality conditions (25)–(27) and condition ($A_k$) with some $k\in \{0,1,2\}$. Then, there exist $\varepsilon _k,\kappa _k > 0$ such that:
  38 $$\begin{aligned} J({\bar{u}}) + \frac{\kappa _k}{2}\Vert y_u - y_{{\bar{u}}}\Vert ^{k}_{L^2(Q)}\Vert u - {\bar{u}}\Vert _{L^1(Q)}^{2-k} \le J(u) \end{aligned}$$
  for all $$u \in \mathcal {U}\text { such that } \Vert y_u - y_{{\bar{u}}}\Vert _{L^\infty (Q)} < \varepsilon _k$$.
2. Let $m\in \mathbb {R}$ and let ${\bar{u}} \in \mathcal {U}$ satisfy the optimality conditions (25)–(27) and condition ($B_k$) with some $k\in \{0,1,2\}$. Then, there exist $\varepsilon _k,\kappa _k > 0$ such that (38) holds for all $u \in \mathcal {U}\text { such that } \Vert u - {\bar{u}}\Vert _{L^1(Q)} < \varepsilon _k$.

Before presenting a proof of Theorem 9, we establish some technical results. The following lemma was proved for various types of objective functionals, see e.g. [10, Lemma 6], [9, Lemma 3.11]. Nevertheless, our objective functional is more general, therefore we present in Appendix A an adapted proof.

#### Lemma 10

Let ${\bar{u}} \in \mathcal {U}$. The following holds.

1. Let $m=0$. For every $\rho > 0$ there exists $\varepsilon > 0$ such that
  39 $$\begin{aligned} \vert [J''({\bar{u}} + \theta (u - {\bar{u}})) - J''({\bar{u}})](u-{\bar{u}})^2\vert \le \rho \Vert z_{{\bar{u}},u-{\bar{u}}}\Vert ^2_{L^2(Q)} \end{aligned}$$
  for all $u \in \mathcal {U}$ with $\Vert y_u - y_{{\bar{u}}}\Vert _{L^\infty (Q)} < \varepsilon$ and $\theta \in [0,1]$.
2. Let $m\in \mathbb {R}$. For every $\rho > 0$ there exists $\varepsilon > 0$ such that (39) holds for all $u \in \mathcal {U}$ with $\Vert u - {\bar{u}}\Vert _{L^1( Q)} < \varepsilon$ and $\theta \in [0,1]$.

For the assumptions with $k\in \{0,1\}$, we need the subsequent corollary, which is also given in Appendix A.

#### Lemma 11

Let ${\bar{u}} \in \mathcal {U}$ and let $m=0$. Then

1. For every $\rho > 0$ there exists $\varepsilon > 0$ such that
  40 $$\begin{aligned} \vert [J''({\bar{u}} + \theta (u - {\bar{u}})) - J''({\bar{u}})](u-{\bar{u}})^2\vert \le \rho \Vert z_{{\bar{u}},u-{\bar{u}}}\Vert _{L^2(Q)}\Vert u-{\bar{u}}\Vert _{L^1(Q)} \end{aligned}$$
  for all $u \in {\mathcal {U}}$ with $\Vert y_u - y_{{\bar{u}}}\Vert _{L^\infty (Q)}< \varepsilon$ and for all $\theta \in [0,1]$.
2. For every $\rho > 0$ there exists $\varepsilon > 0$ such that
  41 $$\begin{aligned} \vert [J''({\bar{u}} + \theta (u - {\bar{u}})) - J''({\bar{u}})](u-{\bar{u}})^2\vert \le \rho \Vert u-{\bar{u}}\Vert _{L^1(Q)}^2 \end{aligned}$$
  for all $u \in \mathcal {U}$ with $\Vert y_u - {\bar{y}}\Vert _{L^\infty (Q)} < \varepsilon$ and for all $\theta \in [0,1]$.

The same assertions hold true for any $m \in \mathbb {R}$ with the inequality $\Vert y_u - y_{{\bar{u}}}\Vert _{L^\infty (Q)} < \varepsilon$ replaced with $\Vert u-{\bar{u}}\Vert _{L^1(Q)} < \varepsilon$.

The next lemma claims that Assumption 3 implies a growth similar to (38) of the first derivative of the objective functional in a neighborhood of ${\bar{u}}$.

#### Lemma 12

Let ${\bar{u}} \in \mathcal {U}$. The following claims are fulfilled.

1. Let $m=0$ and ${\bar{u}}$ satisfy condition $(A_k)$, for some $k\in \{0,1,2\}$. Then, there exist ${\bar{\alpha }}_k,{\bar{\gamma }}_k > 0$ such that
  42 $$\begin{aligned} J'(u)(u - {\bar{u}}) \ge {\bar{\gamma }}_k\Vert z_{{\bar{u}},u - {\bar{u}}}\Vert ^{k}_{L^2(Q)}\Vert u - {\bar{u}}\Vert ^{2-k}_{L^1(Q)} \end{aligned}$$
  for every $$u \in \mathcal {U}\text { with } \Vert y_u -y_{{\bar{u}}}\Vert _{L^\infty (Q)} < {\bar{\alpha }}_k$$.
2. Let $m\in \mathbb {R}$ and let ${\bar{u}}$ satisfy condition $(B_k)$ for some $k\in \{0,1,2\}$. Then, there exist ${\bar{\alpha }}_k,{\bar{\gamma }}_k > 0$ such that (42) holds for every $u \in \mathcal {U}$ with
  $$\Vert u -{\bar{u}}\Vert _{L^1(Q)} < {\bar{\alpha }}_k$$.

#### Proof

Since *J* is of class $C^2$ we can use the mean value theorem to infer the existence of a measurable function $\theta :Q\rightarrow [0,1]$ such that

$$\begin{aligned} J'(u)(u - {\bar{u}})-J'({\bar{u}})(u - {\bar{u}}) =J''({\bar{u}} + \theta (u - {\bar{u}}))(u-{\bar{u}})^2. \end{aligned}$$

Select $k \in \{0,1,2\}$ such that condition $(A_k)$ is satisfied, we infer the existence of positive constants $\gamma _k$ and $\alpha _k$ such that

$$\begin{aligned} J'(u)(u - {\bar{u}})&=J'({\bar{u}})(u - {\bar{u}}) + J''({\bar{u}})(u - {\bar{u}})^2\\&\quad +[J'(u)(u - {\bar{u}})-J'({\bar{u}})(u - {\bar{u}}) - J''({\bar{u}})(u - {\bar{u}})^2]\\&\ge \gamma _k\Vert z_{{\bar{u}},u - {\bar{u}}}\Vert ^{k}_{L^2(Q)}\Vert u - {\bar{u}}\Vert ^{2-k}_{L^1(Q)}\\&\quad -\vert [J''({\bar{u}} + \theta (u - {\bar{u}})) - J''({\bar{u}})](u-{\bar{u}})^2\vert , \end{aligned}$$

for all $u\in {\mathcal {U}}$ with $\Vert y_u-y_{{\bar{u}}}\Vert _{L^\infty (Q)}<\alpha _k$. Using Lemma 10, we obtain that

$$\begin{aligned} J'(u)(u - {\bar{u}})&\ge (\gamma _k-\rho _k)\Vert z_{{\bar{u}},u - {\bar{u}}}\Vert ^{k}_{L^2(Q)}\Vert u - {\bar{u}}\Vert ^{2-k}_{L^1(Q)} \end{aligned}$$

for all $u\in {\mathcal {U}}$ with $\Vert y_u-y_{{\bar{u}}}\Vert _{L^\infty (Q)}<{\bar{\alpha }}_k$ and ${\bar{\alpha }}_k:=\min \{\alpha _k,\varepsilon _k \}$, where $\varepsilon _k>0$ is chosen such that ${\bar{\gamma }}_k:=\gamma _k-\rho _k>0$. This proves the first claim of the lemma. Using the last statement of Lemma 11 concerning the general case $m\in \mathbb {R}$ and the estimate

$$\begin{aligned} \Vert y_u - y_{{\bar{u}}}\Vert _{L^\infty (Q)}\le C_r(2M_{{\mathcal {U}}})^{\frac{r-1}{r}}\Vert u - {\bar{u}}\Vert _{L^1(Q)}^{\frac{1}{r}} \end{aligned}$$

we obtain the second claim. $\square$

Finally, we conclude this subsection with the proof of Theorem 9.

#### Proof of Theorem 9

Using the Taylor expansion and the first order optimality condition satisfied by ${\bar{u}}$ we have

$$\begin{aligned} J(u)&= J({\bar{u}}) + J'({\bar{u}})(u - {\bar{u}}) + \frac{1}{2}J''(u_\theta )(u - {\bar{u}})^2\\&\ge J({\bar{u}}) + \frac{1}{2} J'({\bar{u}})(u - {\bar{u}}) + \frac{1}{2}J''(u_\theta )(u - {\bar{u}})^2 \end{aligned}$$

where $u_\theta :={\bar{u}}+\theta (u-{\bar{u}})$ for a measurable function $\theta : Q \rightarrow [0,1]$. We select $k\in \{0,1,2\}$ such that the corresponding condition in Assumption 3 is satisfied. Then we continue the last inequality, using that, according to the condition, there exist positive $\alpha _k,\gamma _k$ such that (38) holds:

$$\begin{aligned} J(u)&\ge J({\bar{u}}) + \frac{1}{2}[J'({\bar{u}})(u - {\bar{u}}) + J''({\bar{u}})(u - {\bar{u}})^2] + \frac{1}{2}[J''(u_\theta ) - J''({\bar{u}})](u - {\bar{u}})^2]\\&\ge J({\bar{u}}) + \frac{\gamma _k}{2} \Vert z_{{\bar{u}},u - {\bar{u}}}\Vert ^k_{L^2(Q)}\Vert u - {\bar{u}}\Vert ^{2-k}_{L^1(Q)} - \frac{1}{2} \big \vert [J''(u_\theta ) - J''({\bar{u}})](u - {\bar{u}})^2 \big \vert \end{aligned}$$

for all $u\in {\mathcal {U}}$ with either $\Vert y_u-y_{{\bar{u}}} \Vert _{L^\infty (Q)}<\alpha _k$ or $\Vert u - {\bar{u}} \Vert _{L^1(Q)}<\alpha _k$, depending on the chosen condition $(A_k)$ or $(B_k)$. Let $m=0$, by Lemma 10 or Lemma 11 (depending on the condition) there exist $\varepsilon >0$ and ${\bar{\gamma }}_k<\gamma _k$ such that

$$\begin{aligned} \vert [J''(u_\theta ) - J''({\bar{u}})](u - {\bar{u}})^2\vert \le {\bar{\gamma }}_k\Vert z_{{\bar{u}},u - {\bar{u}}}\Vert ^k_{L^2(Q)}\Vert u - {\bar{u}}\Vert ^{2-k}_{L^1(Q)} \end{aligned}$$

for every $u \in \mathcal {U}$ with $\Vert y_u - y_{{\bar{u}}}\Vert _{L^\infty (Q)} <\varepsilon$. We may choose ${\bar{\alpha }}_k>0$ and ${\bar{\gamma }}_k>0$ according to Lemma 12 and depending on the chosen condition therein. Inserting this estimate in the above expression and applying (18) gives

$$\begin{aligned} J(u)&\ge J({\bar{u}}) + \frac{1}{2}(\gamma _k-{\bar{\gamma }}_k)\Vert z_{{\bar{u}},u - {\bar{u}}}\Vert ^k_{L^2(Q)}\Vert u - {\bar{u}}\Vert ^{2-k}_{L^1(Q)}\\&\ge J({\bar{u}}) + \frac{3(\gamma _k-{\bar{\gamma }}_k)}{4}\Vert y_u - y_{{\bar{u}}}\Vert ^{k}_{L^2(Q)}\Vert u - {\bar{u}}\Vert ^{2-k}_{L^1(Q)}, \end{aligned}$$

for all $u\in {\mathcal {U}}$ with $\Vert y_u-y_{{\bar{u}}}\Vert _{L^\infty (Q)}<\min \{\varepsilon ,{\bar{\alpha }}_k\}$ and condition $(A_k)$ follows. For condition $(B_k)$, we use that

$$\begin{aligned} \Vert y_u - y_{{\bar{u}}}\Vert _{L^\infty (Q)}\le C_r (2M_{{\mathcal {U}}})^{\frac{r-1}{r}}\Vert u-{\bar{u}}\Vert _{L^1(Q)}^{\frac{1}{r}} \end{aligned}$$

to apply Lemma 10 or Lemma 11 depending on $k\in \{0,1,2\}$. Finally, for $m \in \mathbb {R}$ and under $(B_k)$ the claim follows by the above arguments applying Lemma 10 or Lemma 11 depending on $k\in \{0,1,2\}$. $\square$

### Some equivalence results for the assumptions on cones

In this subsection we show that some of the items in Assumption 3 can be formulated equivalently on the cones $D^\tau _{{\bar{u}}}$ or $C^{\tau }_{{\bar{u}}}$ respectively. This applies to ($B_k$) or to ($A_k$) depending on whether the objective functional explicitly depends on the control or not. The results in this subsection are important to compare the conditions introduced in Assumption 3 with other conditions in the literature. We need the next lemma, the proof of which uses a result from [7].

#### Lemma 13

Let ${\bar{u}}\in {\mathcal {U}}$ satisfy the first order optimality condition (25)-(27) and let $u\in {\mathcal {U}}$ be given. For any positive number $\tau$, we define

$$\begin{aligned} v:=\left\{ \begin{array}{lll} 0 &{}\text { on } &{}[ \ \vert \frac{\partial {\bar{H}}}{\partial u}\vert >\tau \ ],\\ u-{\bar{u}} &{}\text { else},&{} \end{array}\right. \end{aligned}$$

and $w:=u-{\bar{u}}-v$. Let $\varepsilon >0$ be given. Then there exists a positive constant *C* such that

43 $$\begin{aligned} \max \left\{ \Vert z_{{\bar{u}},w}\Vert _{L^{\infty }(Q)}, \ \Vert z_{{\bar{u}},v}\Vert _{L^{\infty }(Q)}\right\} <C\max \left\{ \varepsilon ,\varepsilon ^{\frac{1}{r}}\right\} \end{aligned}$$

for all $u \in {\mathcal {U}} \text { with }\Vert u-{\bar{u}}\Vert _{L^{1}(Q)}<\varepsilon$. If additionally $\varepsilon$ is such that (18) holds. and the control does not appear explicitly in (1) (that is, $m=g=0$ in (4)), then (43) holds for all $u\in {\mathcal {U}}$ such that $u-{\bar{u}} \in G^\tau _{{\bar{u}}}$ and $\Vert z_{{\bar{u}},u-{\bar{u}}}\Vert _{L^{\infty }(Q)}<\varepsilon$.

#### Proof

We define ${\tilde{u}}, {\hat{u}}\in {\mathcal {U}}$ by

$$\begin{aligned} {\tilde{u}}:=\left\{ \begin{array}{lll}\displaystyle {\bar{u}} &{}\text { on}&{} [\ \vert \frac{\partial {\bar{H}}}{\partial u}\vert>\tau \ ],\\ u &{}\text { else.}&{}\end{array}\right. \ \ {\hat{u}}:= \left\{ \begin{array}{lll}\displaystyle u &{}\text { on}&{} [\ \vert \frac{\partial {\bar{H}}}{\partial u}\vert > \tau \ ],\\ {\bar{u}} &{}\text { else.}\end{array}\right. \end{aligned}$$

Observe that $v={\tilde{u}}-{\bar{u}}$, $w={\hat{u}}-{\bar{u}}$ and $u-{\bar{u}} =v+w$. It is trivial by construction that

$$\begin{aligned} \{\Vert v\Vert _{L^1(Q)},\ \Vert w\Vert _{L^1(Q)}\}\le \Vert u-{\bar{u}}\Vert _{L^1(Q)}. \end{aligned}$$

On the other hand, by (18), $\Vert z_{{\bar{u}},u-{\bar{u}}}\Vert _{L^{\infty }(Q)}<\varepsilon$ implies $\Vert y_{u}-y_{{\bar{u}}}\Vert _{L^{\infty }(Q)}<2\varepsilon$. If $m,g=0$, we can argue as in [7] using $u-{\bar{u}} \in G^\tau _{{\bar{u}}}$ and the definition of *w*, to estimate

44 $$\begin{aligned} \tau \Vert w\Vert _{L^1(Q)}&\le J'({\bar{u}})(u-{\bar{u}})\le \tau \Vert z_{{\bar{u}},u-{\bar{u}}} \Vert _{L^{1}(Q)}. \end{aligned}$$

Thus by Theorem 1, (16), and with $M:= C_r (2M_{{\mathcal {U}}})^{\frac{r-1}{r}}$,

$$\begin{aligned} \Vert z_{{\bar{u}},w} \Vert _{L^\infty (Q)}\le \left\{ \begin{array}{lll} M\Vert z_{{\bar{u}},u-{\bar{u}}}\Vert _{L^\infty (Q)}^{\frac{1}{r}} &{}\text { if }&{}m,g=0,\ u-{\bar{u}} \in G^\tau _{{\bar{u}}},\\ M\Vert u-{\bar{u}} \Vert _{L^1(Q)}^{\frac{1}{r}} &{}\text { else.}&{} \end{array}\right. \end{aligned}$$

For $z_{{\bar{u}}, v}$ we estimate with $C:=2(M+1)$

$$\begin{aligned} \Vert z_{{\bar{u}},v} \Vert _{L^\infty (Q)}&\le \Vert z_{{\bar{u}},v+w} \Vert _{L^\infty (Q)}+\Vert -z_{{\bar{u}},w} \Vert _{L^\infty (Q)} \le C\max \left\{ \varepsilon ,\varepsilon ^{\frac{1}{r}}\right\} . \end{aligned}$$

In the second case the estimate holds trivially. $\square$

Now we continue with the equivalence properties.

#### Corollary 14

For $k\in \{0,2\}$, condition $(B_k)$ is equivalent to the following condition (${\bar{B}}_{k}$): there exist positive constants $\alpha _k,\gamma _k$ and $\tau$ such that

45 $$\begin{aligned} J'({\bar{u}})(u - {\bar{u}}) + J''({\bar{u}})(u - {\bar{u}})^2 \ge \gamma _k\Vert z_{{\bar{u}},u - {\bar{u}}}\Vert ^{k}_{L^2(Q)}\Vert u - {\bar{u}}\Vert ^{2-k}_{L^1(Q)}, \end{aligned}$$

for all $u \in \mathcal {U}$ for which $(u-{\bar{u}} )\in D_{{\bar{u}}}^{\tau }\text { and } \Vert u-{\bar{u}} \Vert _{L^{1}(Q)} < \alpha _k$.

#### Proof

Let $k\in \{0,2\}$. If ($B_k$) holds then (${\bar{B}}_{k}$) is obviously also fulfilled. Now let (${\bar{B}}_{k}$) hold. The numbers ${\tilde{\alpha }}_k$ and ${\tilde{\gamma }}_k$ will be chosen later so that assumption $(B_k)$ will hold with these numbers. For now we only require that $0< {\tilde{\alpha }}_k < \alpha _k$. Choose an arbitrary $u \in \mathcal {U}$ with $\Vert u-{\bar{u}} \Vert _{L^{1}(Q)} < {\tilde{\alpha }}_k$. We only need to prove (37) in the case $u-{\bar{u}}\notin D^{\tau }_{{\bar{u}}}$. Take *v* and *w* as defined in Lemma 13. Clearly by definition $v\in D^\tau _{{\bar{u}}}$. As a direct consequence of (22)-(23) and Assumption 1 and 2 there exists a positive constant *M* such that

46 $$\begin{aligned} \vert J''({\bar{u}})(w)^2\vert&\le M \Vert z_{{\bar{u}},w}\Vert _{L^\infty (Q)} \Vert w\Vert _{L^1(Q)}, \end{aligned}$$

47 $$\begin{aligned} \vert J''({\bar{u}})(w,v)\vert&\le M \Vert z_{{\bar{u}}, v}\Vert _{L^\infty (Q)} \Vert w\Vert _{L^1(Q)}. \end{aligned}$$

Since ${\tilde{\alpha }}_k < \alpha _k$ and $v\in D^\tau _{{\bar{u}}}$ we may apply (45) with *v* instead of $u-{\bar{u}}$. Using also (46) and (47), we estimate

$$\begin{aligned}&J'({\bar{u}})(u - {\bar{u}}) + J''({\bar{u}})(u - {\bar{u}})^2=J'({\bar{u}})(v+w) + J''({\bar{u}})(v+w)^2\\&\quad \ge J'({\bar{u}})(v)+J'({\bar{u}})(w)+ J''({\bar{u}})(v)^2+J''({\bar{u}})(w)^2+2J''({\bar{u}})(w,v)\\&\quad \ge \gamma _k \Vert z_{{\bar{u}}, v} \Vert _{L^2(Q)}^{k} \Vert v\Vert _{L^1(Q)}^{2-k}+\tau \Vert w\Vert _{L^1(Q)}\\&\quad -3M(\Vert z_{{\bar{u}}, w}\Vert _{L^\infty (Q)}+\Vert z_{{\bar{u}}, v}\Vert _{L^\infty (Q)})\Vert w \Vert _{L^1(Q)}\\&\quad \ge \gamma _k \Vert z_{{\bar{u}}, v} \Vert _{L^2(Q)}^{k} \Vert v\Vert _{L^1(Q)}^{2-k}+\frac{\tau }{2} \Vert w\Vert _{L^1(Q)}. \end{aligned}$$

In the last inequality we use that by choosing ${\tilde{\alpha }}_k>0$ sufficiently small we may ensure that

$$\begin{aligned}&\tau -3M(\Vert z_{{\bar{u}}, w}\Vert _{L^\infty (Q)}+\Vert z_{{\bar{u}}, v}\Vert _{L^\infty (Q)})\\&\quad \ge \tau -3MC\max \left\{ {\tilde{\alpha }},{\tilde{\alpha }}^{\frac{1}{r}}\right\} \ge \frac{\tau }{2}. \end{aligned}$$

This holds because by Lemma 13, there exists a positive constant *C* such that

48 $$\begin{aligned} \max \left\{ \Vert z_{{\bar{u}},w}\Vert _{L^{\infty }(Q)}, \ \Vert z_{{\bar{u}},v}\Vert _{L^{\infty }(Q)}\right\} \le C\max \left\{ {\tilde{\alpha }}_2,{\tilde{\alpha }}_2^{\frac{1}{r}}\right\} . \end{aligned}$$

Further, we use that $\Vert u-{\bar{u}}\Vert _{L^1(Q)}<2M_{{\mathcal {U}}}$ for all $u\in {\mathcal {U}}$, (8) and (10) in Lemma 2 for $s=1$, to estimate

49 $$\begin{aligned} \Vert z_{{\bar{u}}, w}\Vert _{L^2(Q)}^2\le \Vert z_{{\bar{u}}, w}\Vert _{L^{\infty }(Q)} \Vert z_{{\bar{u}}, w} \Vert _{L^1(Q)} \le 2C_\infty C_r M_{{\mathcal {U}}}\vert Q \vert ^{\frac{1}{r}}\Vert w\Vert _{L^1(Q)} \end{aligned}$$

By this, we find

50 $$\begin{aligned} \Vert w\Vert _{L^1(Q)}\ge \left\{ \begin{array}{l} \frac{1}{2M_{{\mathcal {U}}}\vert Q\vert }\Vert w \Vert _{L^1(Q)}^2,\\ \frac{1}{2C_\infty C_r M_{{\mathcal {U}}}\vert Q \vert ^{\frac{1}{r}}} \Vert z_{{\bar{u}}, w}\Vert _{L^2(Q)}^2. \end{array}\right. \end{aligned}$$

Finally, we make the estimations for the different cases.

For $$k=0$$:

$$\begin{aligned} J'({\bar{u}})(u - {\bar{u}}) + J''({\bar{u}})(u - {\bar{u}})^2&\ge \gamma _0 \Vert v\Vert _{L^1(Q)}^{2}+\frac{\tau }{2M_{{\mathcal {U}}}\vert Q\vert } \Vert w\Vert _{L^1(Q)}^2\\&\ge \min \Big \{\gamma _0, \frac{\tau }{2M_{{\mathcal {U}}}\vert Q\vert }\Big \}(\Vert v\Vert _{L^1(Q)}^{2}+\Vert w\Vert _{L^1(Q)}^2)\\&\ge \frac{1}{2}\min \Big \{\gamma _0,\frac{\tau }{2M_{{\mathcal {U}}}\vert Q\vert }\Big \}(\Vert u-{\bar{u}}\Vert _{L^1(Q)}^{2}. \end{aligned}$$

For $$k=2$$:

$$\begin{aligned} J'({\bar{u}})(u - {\bar{u}})&+ J''({\bar{u}})(u - {\bar{u}})^2 \ge \gamma _2 \Vert z_{{\bar{u}}, v} \Vert _{L^2(Q)}^{2}+\frac{\tau }{2} \Vert w\Vert _{L^1(Q)}\\&\ge \min \Big \{\gamma _2, \frac{\tau }{2C_\infty C_r M_{{\mathcal {U}}}\vert Q \vert ^{\frac{1}{r}}}\Big \}( \Vert z_{{\bar{u}}, v}\Vert _{L^2(Q)}^{2} +\Vert z_{{\bar{u}}, w}\Vert _{L^2(Q)}^2)\\&\ge \frac{1}{2}\min \Big \{\gamma _2, \frac{\tau }{2C_\infty C_r M_{{\mathcal {U}}}\vert Q \vert ^{\frac{1}{r}}}\Big \}\Vert z_{{\bar{u}}, u-{\bar{u}}}\Vert _{L^2(Q)}^2. \end{aligned}$$

This proves that (37) is satisfied with an appropriate number ${\tilde{\gamma }}_k$. $\square$

If the control does not appear explicitly in the objective functional, we obtain a stronger result.

#### Corollary 15

Let $m,g=0$. Then condition $(A_2)$ is equivalent to the following condition (${\bar{A}}_{2}$): there exist positive constants $\alpha _2,\gamma _2,\tau$ such that

51 $$\begin{aligned} J'({\bar{u}})(u - {\bar{u}}) + J''({\bar{u}})(u - {\bar{u}})^2 \ge \gamma _2\Vert z_{{\bar{u}},u - {\bar{u}}}\Vert ^{2}_{L^2(Q)} \end{aligned}$$

for all $u \in \mathcal {U}$ for which $(u-{\bar{u}} )\in C^{\tau }_{{\bar{u}}}$ and $\Vert y_u-y_{{\bar{u}}}\Vert _{L^\infty (Q)}<\alpha _2$.

#### Proof

It is obvious that $(A_2)$ implies $({\bar{A}}_{2})$. For the reverse, if $u-{\bar{u}} \in C^{\tau }_{{\bar{u}}}$ the estimate holds trivially. We need to consider the cases $u-{\bar{u}}\notin G^{\tau }_{{\bar{u}}}$ and $u-{\bar{u}}\notin D^{\tau }_{{\bar{u}}}\text { with }u-{\bar{u}}\in G^{\tau }_{{\bar{u}}}$. For the first, we argue as follows. Since $u-{\bar{u}}\notin G^{\tau }_{{\bar{u}}}$ it holds

$$\begin{aligned} J'({\bar{u}})(u-{\bar{u}})+J''({\bar{u}})(u-{\bar{u}})&> \frac{\tau }{2} \Vert z_{{\bar{u}},u-{\bar{u}}}\Vert _{L^1(Q)}\ge \frac{\tau }{4C_rM_{{\mathcal {U}}}\vert Q\vert ^{\frac{1}{r}}}\Vert z_{{\bar{u}},u-{\bar{u}}} \Vert _{L^2(Q)}^2. \end{aligned}$$

For the second case $u-{\bar{u}}\in G^{\tau }_{{\bar{u}}}$ and $u-{\bar{u}}\notin D^{\tau }_{{\bar{u}}}$, let ${\tilde{\alpha }}>0$ be smaller than $\alpha _2$, so that (51) and the prerequisite of Lemma 13 is satisfied. We define *w*, *v* as in Lemma 13. By the choice of $\alpha _2$, Lemma 13 gives the existence of a positive constant *C* such that $\Vert z_{{\bar{u}},u-{\bar{u}}}\Vert _{L^\infty }<\alpha _2$ implies

$$\begin{aligned} \max \left\{ \Vert z_{{\bar{u}},w}\Vert _{L^{\infty }(Q)}, \ \Vert z_{{\bar{u}},v}\Vert _{L^{\infty }(Q)}\right\} <C\max \left\{ \alpha _2,\alpha _2^{\frac{1}{r}}\right\} . \end{aligned}$$

Now we can proceed by the same arguments as in Corollary 14

$$\begin{aligned} J'({\bar{u}})(u - {\bar{u}}) + J''({\bar{u}})(u - {\bar{u}})^2&=J'({\bar{u}})(v+w) + J''({\bar{u}})(v+w)^2\\&\ge \gamma _2 \Vert z_{{\bar{u}}, v} \Vert _{L^2(Q)}^{2}+\frac{\tau }{2} \Vert w\Vert _{L^1(Q)}. \end{aligned}$$

Finally, we use (50) to obtain that

$$\begin{aligned} J'({\bar{u}})(u - {\bar{u}})&+ J''({\bar{u}})(u - {\bar{u}})^2 \ge \gamma _2 \Vert z_{{\bar{u}}, v} \Vert _{L^2(Q)}^{2}+\frac{\tau }{2C_\infty C_r M_{{\mathcal {U}}}\vert Q \vert ^{\frac{1}{r}}} \Vert w\Vert _{L^1(Q)}\\&\ge \min \Big \{\gamma _2, \frac{\tau }{2C_\infty C_r M_{{\mathcal {U}}}\vert Q \vert ^{\frac{1}{r}}}\Big \}( \Vert z_{{\bar{u}}, v} \Vert _{L^2(Q)}^{2} +\Vert z_{{\bar{u}}, w}\Vert _{L^2(Q)}^2)\\&\ge \min \Big \{\gamma _2, \frac{\tau }{2C_\infty C_r M_{{\mathcal {U}}}\vert Q \vert ^{\frac{1}{r}}}\Big \}( \Vert z_{{\bar{u}}, u-{\bar{u}}} \Vert _{L^2(Q)}^{2}, \end{aligned}$$

for all $(u-{\bar{u}} )\in C^{\tau }_{{\bar{u}}}$ with $\Vert y_u-y_{{\bar{u}}}\Vert _{L^\infty (Q)}<\alpha _2$. $\square$

Although we can not prove a similar equivalence property for the condition $(A_1)$ in Assumption 3, below we show that it is implied by the structural assumption (34) and a second order sufficient condition.

#### Proposition 16

Let $m,g=0$. Then the structural assumption (34) and the second order sufficient condition (33) (for $G=C^\tau _{{\bar{u}}}$) imply condition $(A_1)$.

#### Proof

Let *u* be an arbitrary element of ${{\mathcal {U}}}$. We consider several cases.

**1.** If $u-{\bar{u}} \in C^\tau _{{\bar{u}}}$ we employ the structural assumption (34) that implies the existence of a positive constant $\gamma _1$ such that

52 $$\begin{aligned} J'({\bar{u}})(u-{\bar{u}})\ge \gamma _1 \Vert u-{\bar{u}}\Vert _{L^1(Q)}^2 \ \ \ \text { for all } u\in {\mathcal {U}}. \end{aligned}$$

Further since $u-{\bar{u}} \in C^\tau _{{\bar{u}}}$, by the second order sufficient optimality condition (33) there exists a positive constant $\gamma _2$ such that

53 $$\begin{aligned} J''({\bar{u}})(u-{\bar{u}})^2\ge \gamma _2 \Vert z_{{\bar{u}},u-{\bar{u}}}\Vert _{L^2(Q)}^2. \end{aligned}$$

Altogether, using the inequality $a^2+b^2 \ge 2 a b$ we obtain

54 $$\begin{aligned} J'({\bar{u}})(u-{\bar{u}})+J''({\bar{u}})(u-{\bar{u}})^2\ge 2\sqrt{\gamma _1\gamma _2}\Vert z_{{\bar{u}},u-{\bar{u}}}\Vert _{L^2(Q)}\Vert u-{\bar{u}}\Vert _{L^1(Q)}, \end{aligned}$$

which implies $(A_1)$ with $\gamma = 2\sqrt{\gamma _1\gamma _2}$ and any $\alpha _1 > 0$.

**2.** Now we consider the case where $u-{\bar{u}} \notin G^\tau _{{\bar{u}}}$. it holds

55 $$\begin{aligned} J'({\bar{u}})(u-{\bar{u}})>\tau \Vert z_{{\bar{u}},u-{\bar{u}}}\Vert _{L^1(Q)}. \end{aligned}$$

On the other hand by the structural assumption (34) we have (52). Further there exists a positive constant *M* such that there exists a $u \in {\mathcal {U}}$

56 $$\begin{aligned} \vert J''({\bar{u}})(u-{\bar{u}})^2\vert \le M\Vert z_{{\bar{u}},u-{\bar{u}}}\Vert _{L^\infty (Q)}\Vert z_{{\bar{u}},u-{\bar{u}}}\Vert _{L^1(Q)}. \end{aligned}$$

Splitting the first variation into two parts and applying either (52) or (55) we conclude also using (56) and taking $\Vert y_u-y_{{\bar{u}}}\Vert _{L^\infty (Q)}$ sufficently small, such that by (18), $\Vert z_{{\bar{u}},u-{\bar{u}}}\Vert _{L^\infty (Q)}$ is sufficiently small

$$\begin{aligned} J'({\bar{u}})(u-{\bar{u}})+J''({\bar{u}})(u-{\bar{u}})^2&\ge \frac{1}{2}(\gamma _1\Vert u-{\bar{u}}\Vert _{L^1(Q)}^2 +\tau \Vert z_{{\bar{u}},u-{\bar{u}}}\Vert _{L^1(Q)})\\&\quad - M \Vert z_{{\bar{u}},u-{\bar{u}}}\Vert _{L^\infty (Q)}\Vert z_{{\bar{u}},u-{\bar{u}}}\Vert _{L^1(Q)}\\&\ge \frac{1}{2}(\gamma _1\Vert u-{\bar{u}}\Vert _{L^1(Q)}^2+\frac{\tau }{2}\Vert z_{{\bar{u}},u-{\bar{u}}}\Vert _{L^1(Q)}). \end{aligned}$$

Applying the estimate

$$\begin{aligned} \Vert z_{{\bar{u}},u-{\bar{u}}}\Vert _{L^2(Q)}^2&\le \Vert z_{{\bar{u}},u-{\bar{u}}}\Vert _{L^\infty (Q)} \Vert z_{{\bar{u}},u-{\bar{u}}}\Vert _{L^1(Q)}\\&\le 2C_rM_{{\mathcal {U}}}\vert Q\vert ^{\frac{1}{r}}\Vert z_{{\bar{u}},u-{\bar{u}}}\Vert _{L^1(Q)} \end{aligned}$$

and the inequlity $a^2+b^2 \ge 2 a b$, the claim follows.

**3.** Finally, we consider the case $u-{\bar{u}} \in G^\tau _{{\bar{u}}}$ and $u-{\bar{u}} \notin D^\tau _{{\bar{u}}}$. We select *v*, *w* as defined in Lemma 13. By definition $v\in C^\tau _{{\bar{u}}}$. We proceed by splitting the first and second variation accordingly and applying (44),(52), (56) and taking $\Vert z_{{\bar{u}},u-{\bar{u}}}\Vert _{L^\infty (Q)}$ sufficiently small to estimate

$$\begin{aligned}&J'({\bar{u}})(u-{\bar{u}})+J''({\bar{u}})(u-{\bar{u}})^2\\&\quad =J'({\bar{u}})(v)+J'({\bar{u}})(w)+J''({\bar{u}})(v)^2+J'({\bar{u}})(w)^2+2J''({\bar{u}})(v,w)\\&\quad \ge \gamma _1\Vert v\Vert _{L^1(Q)}^2+\frac{\gamma _1}{2}\Vert w\Vert _{L^1(Q)}^2+\frac{\tau }{2}\Vert w\Vert _{L^1(Q)}+ \gamma _2\Vert z_{{\bar{u}},v}\Vert _{L^2(Q)}^2\\&\quad -M\max \left\{ \Vert z_{{\bar{u}},v}\Vert _{L^\infty (Q)},\Vert z_{{\bar{u}},w}\Vert _{L^\infty (Q)}\right\} \Vert z_{{\bar{u}},w}\Vert _{L^1(Q)}\\&\quad \ge 2\sqrt{\frac{\gamma _1^2}{2}}\Vert u-{\bar{u}}\Vert _{L^1(Q)}^2+\gamma _2\Vert z_{{\bar{u}},v}\Vert _{L^2(Q)}^2+\frac{\tau }{4}\Vert w\Vert _{L^1(Q)}. \end{aligned}$$

Then $(A_1)$ follows from the second estimation in (50) and the inequality $a^2+b^2 \ge 2 a b$. $\square$

## Strong metric Hölder subregularity and auxiliary results

We study the strong metric Hölder subregularity property (SMHSr) of the optimality map. This is an extension of the strong metric subregularity property (see, [18, Section 3I] or [15, Section 4]) dealing with Lipschitz stability of set-valued mappings. The SMHSr property is especially relevant to the parabolic setting where Lipschitz stability may fail.

### The optimality mapping

We begin by defining some mappings used to represent the optimality in a more convenient way. This is done analogously to [17, Section 2.1]. Given the initial data $y_0$ in (2), we define the set

57 $$\begin{aligned} D({\mathcal {L}}):=\Big \{ y\in W(0,T)\cap L^{\infty }(Q)\Big \vert \ \Big (\frac{d}{dt}+{\mathcal {A}}\Big )y \in L^{r}(Q), y(\cdot ,0)=y_0 \Big \}. \end{aligned}$$

To shorten notation, we define ${\mathcal {L}}:D({\mathcal {L}})\rightarrow L^{r}(Q)$ by ${\mathcal {L}}:=\frac{d}{dt}+{\mathcal {A}}$. Additionally, we define the mapping ${\mathcal {L}}^*:D({\mathcal {L}}^*)\rightarrow L^{r}(Q)$ by ${\mathcal {L}}^*:=(-\frac{d}{dt}+{\mathcal {A}}^*)$, where

$$\begin{aligned} D({\mathcal {L}}^*):=\Big \{ p\in W(0,T)\cap L^{\infty }(Q)\Big \vert \Big (-\frac{d}{dt}+{\mathcal {A}}^*\Big )p \in L^{r}(Q), p(\cdot ,T)=0 \Big \}. \end{aligned}$$

With the mappings ${\mathcal {L}}$ and ${\mathcal {L}}^*$, we recast the semilinear state Eq. (2) and the linear adjoint equation (26) in a short way:

$$\begin{aligned} {\mathcal {L}} y= & {} u-f(\cdot ,y)\\ {\mathcal {L}}^* p= & {} L_y(\cdot ,y_u,u)-pf_y(\cdot ,y_u)=\frac{\partial H}{\partial y}(\cdot ,y_u,p,u). \end{aligned}$$

The normal cone to the set $\mathcal {U}$ at $u \in L^1(Q)$ is defined in the usual way:

$$\begin{aligned} N_{\mathcal {U}}(u):= \left\{ \begin{array}{cl} \big \{\nu \in L^{\infty }(Q)\big \vert \ \int _Q \nu (v-u)\,\textrm{d}x\,\textrm{d}t\le 0 \ \ \forall v \in {\mathcal {U}} \big \} &{} \text{ if } u \in \mathcal {U}, \\ \emptyset &{} \text{ if } u \not \in \mathcal {U}. \end{array} \right. \end{aligned}$$

The first order necessary optimality condition for problem (1)-(3) in Theorem 7 can be recast as

58 $$\begin{aligned} \left\{ \begin{array}{cll} 0&{}=&{}{\mathcal {L}} y+f(\cdot ,y)-u,\\ 0&{}=&{}{\mathcal {L}}^*p- \frac{\partial H}{\partial y}(\cdot ,y,p,u),\\ 0&{}\in &{} H_{u}(\cdot ,y,p) + N_{{\mathcal {U}}}(u). \end{array} \right. \end{aligned}$$

For (58) to make sense, a solution (*y*, *p*, *u*) must satisfy $y\in D({\mathcal {L}})$, $p\in D({\mathcal {L}}^*)$ and $u\in {\mathcal {U}}$. For a local solution ${\bar{u}}\in {\mathcal {U}}$ of problem (1)-(3), by Theorem 7, the triple $(y_{{\bar{u}}},p_{{\bar{u}}},{\bar{u}})$ is a solution of (58). We define the sets

59 $$\begin{aligned} {\mathcal {Y}}:=D({\mathcal {L}})\times D({\mathcal {L}}^*)\times {\mathcal {U}}\quad \text {and}\quad {\mathcal {Z}}:= L^2(Q)\times L^2(Q)\times L^\infty (Q), \end{aligned}$$

and consider the set-valued mapping $\Phi :{\mathcal {Y}}\twoheadrightarrow {\mathcal {Z}}$ given by

60 $$\begin{aligned} \Phi \left( \begin{array}{c} y \\ p \\ u \end{array} \right) :=\left( \begin{array}{c} \mathcal {L}y + f(\cdot ,y)-u \\ {\mathcal {L}}^*p- \frac{\partial H}{\partial y}(\cdot ,y,p,u) \\ \frac{\partial H}{\partial u}(\cdot ,y,p,u)+ N_{{\mathcal {U}}}(u) \end{array} \right) . \end{aligned}$$

With the abbreviation $\psi := (y,p,u)$, the system (58) can be rewritten as the inclusion $0\in \Phi (\psi )$. Our goal is to study the stability of system (58), or equivalently, the stability of the solutions of the inclusion $0\in \Phi (\psi )$ under perturbations. For elements $\xi ,\eta \in L^r(Q)$ and $\rho \in L^\infty (Q)$ we consider the perturbed system

61 $$\begin{aligned} \left\{ \begin{array}{cll} \xi &{}=&{}\mathcal Ly+f(\cdot ,y) - u,\\ \eta &{}=&{}{\mathcal {L}}^*p-\frac{\partial H}{\partial y}(\cdot ,y,p,u),\\ \rho &{}\in &{}\frac{\partial H}{\partial u}(\cdot ,y,p) + N_{{\mathcal {U}}}(u), \end{array} \right. \end{aligned}$$

which is equivalent to the inclusion $\zeta := (\xi ,\eta ,\rho )\in \Phi (\psi )$.

#### Definition 2

The mapping $\Phi :{\mathcal {Y}}\twoheadrightarrow {\mathcal {Z}}$ is called the *optimality mapping* of the optimal control problem (1)-(3).

#### Theorem 17

For any perturbation $\zeta :=(\xi ,\eta ,\rho )\in L^r(Q)\times L^r(Q)\times L^\infty (Q)$ there exists a triple $\psi := (y,p,u)\in {\mathcal {Y}}$ such that $\zeta \in \Phi (\psi )$.

#### Proof

We consider the optimal control problem

$$\begin{aligned} \min _{u \in {\mathcal {U}}} \Big \{{\mathcal {J}} (u)+\int _Q \eta y \text { dxdt}-\int _Q \rho u \text { dxdt}\Big \}, \end{aligned}$$

subject to

$$\begin{aligned} \left\{ \begin{array}{l} {\mathcal {L}} y+f(x,t,y) = u + \xi \ \text { in }\ Q,\\ y=0 \ \text { on }\ \Sigma ,\ y(\cdot ,0)=y_0 \text { in } \Omega . \end{array} \right. \end{aligned}$$

Under assumptions 1 and 2, we have by standard arguments the existence of a global solution ${\tilde{u}}$. Then ${\tilde{u}}$ and the corresponding state $y_{{\tilde{u}}}$ and adjoint state $p_{{\tilde{u}}}$ satisfy (61). $\square$

Given a metric space $({\mathcal {X}},d_{{\mathcal {X}}})$, we denote by $B_{{\mathcal {X}}}(c,\alpha )$ the closed ball of center $c\in {\mathcal {X}}$ and radius $\alpha >0$. The spaces ${\mathcal {Y}}$ and ${\mathcal {Z}}$, introduced in (59), are endowed with the metrics

62 $$\begin{aligned} d_{{\mathcal {Y}}}(\psi _1,\psi _2)&:=\Vert y_1-y_2\Vert _{L^2(Q)}+\Vert p_1-p_2\Vert _{L^2(Q)}+\Vert u_1-u_2\Vert _{L^1(Q)},\nonumber \\ d_{{\mathcal {Z}}}(\zeta _1,\zeta _2)&:=\Vert \xi _1-\xi _2\Vert _{L^{2}(Q)}+\Vert \eta _1-\eta _2\Vert _{L^{2}(Q)}+ \Vert \rho _1-\rho _2\Vert _{L^\infty (Q)}, \end{aligned}$$

where $\psi _i=(y_i,p_i,u_i)$ and $\zeta _i=(\xi _i,\eta _i,\rho _i)$, $i\in \{1,2\}$. From now on, we denote ${\bar{\psi }}:=(y_{{\bar{u}}},p_{{\bar{u}}},{\bar{u}})$ to simplify notation.

The following extension of the previous theorem can be proved along the lines of [17, Theorem 4.12].

#### Theorem 18

Let condition $(A_0)$ hold. For each $\varepsilon > 0$ there exists $\delta > 0$ such that for every $\zeta \in B_{Z} (0;\delta )$ there exists $\psi \in B_{Y}({\bar{\psi }}; \varepsilon )$ satisfying the inclusion $\zeta \in \Phi (\psi )$.

### Strong metric Hölder subregularity: main result

This subsection contains one of the main results in this paper: estimates of the difference between the solutions of the perturbed system (61) and a reference solution of the unperturbed one, (58), by the size of the perturbations. This will be done using the notion of *strong metric Hölder subregularity* introduced in the next paragraphs.

#### Definition 3

Let ${\bar{\psi }}$ satisfy $0 \in \Phi ({\bar{\psi }})$. We say that the optimality mapping $\Phi :{\mathcal {Y}}\twoheadrightarrow {\mathcal {Z}}$ is *strongly metrically Hölder subregular* (SMHSr) at $({\bar{\psi }},0)$ with exponent $\theta >0$ if there exist positive numbers $\alpha _1,\alpha _2$ and $\kappa$ such that

$$\begin{aligned} d_{{\mathcal {Y}}}(\psi ,{\bar{\psi }})\le \kappa d_{{\mathcal {Z}}}(\zeta ,0)^\theta \end{aligned}$$

for all $\psi \in B_{{\mathcal {Y}}}({\bar{\psi }}\text {; }\alpha _1)$ and $\zeta \in B_{{\mathcal {Z}}}(0; \alpha _2)$ satisfying $\zeta \in \Phi (\psi )$.

Notice that applying the definition with $\zeta = 0$ we obtain that ${\bar{\psi }}$ is the unique solution of the inclusion $0 \in \Phi (\psi )$ in $B_{{\mathcal {Y}}}({\bar{\psi }}; \alpha _1)$. In particular, ${\bar{u}}$ is a strict local minimizer for problem (1)-(3).

In the next assumption we introduce a restriction on the set of admissible perturbations, call it $\Gamma$, which is valid for the remaining part of this section.

#### Assumption 4

For a fixed positive constant $C_{pe}$, the admissible perturbation $\zeta = (\xi ,\eta ,\rho ) \in \Gamma \subset {\mathcal {Z}}$ satisfy the restriction

63 $$\begin{aligned} \Vert \xi \Vert _{L^r(Q)}, \Vert \eta \Vert _{L^r(Q)}\le C_{pe} . \end{aligned}$$

For any $u \in {\mathcal {U}}$ and $\zeta \in \Gamma$ we denote by $(y_u^\zeta , p_u^\zeta ,u)$ a solution of the first two equations in (61). Using (11,12) in Theorem 3 we obtain the existence of a constant $K_y$ such that

64 $$\begin{aligned} \Vert y_u^\zeta \Vert _{L^\infty (Q)} \le K_y \quad \forall u \in {\mathcal {U}} \;\; \forall \zeta \in \Gamma . \end{aligned}$$

Then for every $u \in {{\mathcal {U}}}$, every admissible disturbance $\zeta$, and the corresponding solution *y* of the first equation in (61) it holds that $(y_u^\zeta (x,t),u(x,t)) \in R:= [- K_y, K_y] \times [u_a, u_b]$.

#### Remark 4

We apply the local properties in Assumption 2 to the interval $[-K_y, K_y]$, and denote further by ${\bar{C}}$ a positive constant that majorates the bounds and the Lipschitz constants of *f* and $L_0$ and their first and second derivatives with respect to $y \in [-K_y, K_y]$.

By increasing the constant $K_y$, if necessary, we may also estimate the adjoint state:

65 $$\begin{aligned} \Vert p_u^\zeta \Vert _{L^\infty (Q)} \le K_y (1 + \Vert \eta \Vert _{L^r(Q)}) \quad \forall u \in {\mathcal {U}} \;\; \forall \zeta \in \Gamma . \end{aligned}$$

This follows from Theorem 1 with $\alpha = - \frac{\partial f}{\partial y}(x,t,y_u^\zeta )$ and with $\frac{\partial L}{\partial y}(x,t,y_u^\zeta ,u)$ at the place of *u*.

We need some technical lemmas before stating our main result.

#### Lemma 19

Let $u\in {\mathcal {U}}$ be given and $v, \eta \in L^r(Q)$, $\xi \in L^\infty (Q)$. Consider solutions $y_u, p_u, z_{ u, v}$ and $y_u^\xi , p_u^\eta , z_{ u,v}^\xi$ of the equations

66 $$\begin{aligned} \left\{ \begin{array}{cll} &{}\mathcal Ly+f(\cdot ,y)&{}=u,\\ &{}{\mathcal {L}}^*p-\frac{\partial H}{\partial y}(\cdot ,y_u,p,u)&{}=0,\\ &{}{\mathcal {L}}_0 z+f_y(\cdot ,y_u)z&{}=v. \end{array} \right. \ \left\{ \begin{array}{cll} &{}\mathcal Ly+f(\cdot ,y)&{}=u+\xi ,\\ &{}{\mathcal {L}}^*p-\frac{\partial H}{\partial y}(\cdot ,y_u^\xi ,p,u)&{}=\eta ,\\ &{}{\mathcal {L}}_0 z+f_y(\cdot ,y^\xi _u)z&{}=v. \end{array} \right. \end{aligned}$$

Here, ${\mathcal {L}}_0$ is defined as ${\mathcal {L}}$, but on the domain (57) with $y_0=0$. There exist positive constants $K_s, K_2$ and $R_2$, independent of $\zeta \in \Gamma$, such that the following inequalities hold

67 $$\begin{aligned}&\Vert y^\xi _u - y_u\Vert _{L^2(Q)} \le C_2 \Vert \xi \Vert _{L^2(Q)}, \end{aligned}$$

68 $$\begin{aligned}&\Vert z^\xi _{u,v} - z_{u,v}\Vert _{L^2(Q)} \le K_2 \Vert \xi \Vert _{L^{r}(Q)}\Vert z_{u,v}\Vert _{L^2(Q)}, \end{aligned}$$

69 $$\begin{aligned}&\Vert z^\xi _{u,v} - z_{u,v}\Vert _{L^s(Q)} \le K_s \Vert \xi \Vert _{L^{2}(Q)}\Vert z_{u,v}\Vert _{L^2(Q)}, \end{aligned}$$

70 $$\begin{aligned}&\Vert p^\eta _u - p_u\Vert _{2}\le R_2(\Vert \xi \Vert _{L^2(Q)}+\Vert \eta \Vert _{L^2(Q)}), \end{aligned}$$

where $$C_2 $$ is the constant given in (7) and $$ s\in [1,\frac{n+2}{n})$$.

#### Proof

Subtracting the state equations in (66) and using the mean value theorem we obtain

$$\begin{aligned} \frac{d}{dt}(y^\xi _u - y_u)+\mathcal {A}(y^\xi _u - y_u) + \frac{\partial f}{\partial y}(x,t,y_\theta )(y^\xi _u - y_u) = \xi . \end{aligned}$$

Then, (7) implies (67). To prove (68) we subtract the equations satisfied by $z^\xi _{u,v}$ and $z_{u,v}$ to obtain

$$\begin{aligned}{} & {} \frac{d}{dt}(z^\xi _{u,v} - z_{u,v})+\mathcal {A}(z^\xi _{u,v} - z_{u,v}) + \frac{\partial f}{\partial y}(x,t,y^\xi _u)(z^\xi _{u,v} - z_{u,v})\\{} & {} \quad = \Big [\frac{\partial f}{\partial y}(x,t,y_u) - \frac{\partial f}{\partial y}(x,t,y^\xi _u)\Big ]z_{u,v}. \end{aligned}$$

Now, using (7), the mean value theorem and (63), (64) with regard to Remark 4 we obtain that

$$\begin{aligned}&\Vert z^\xi _{u,v} - z_{u,v}\Vert _{L^2(Q)} \le C_2 \Big \Vert \Big [\frac{\partial f}{\partial y}(x,t,y_u) -\frac{\partial f}{\partial y}(x,t,y^\xi _u)\Big ]z_{u,v}\Big \Vert _{L^2(Q)}\\&\quad \le C_2 \bar{C}\Vert (y^\xi _u - y_u)z_{u,v}\Vert _{L^2(Q)}\le C_2 \bar{C}\Vert y^\xi _u - y_u\Vert _{L^\infty (Q)}\Vert z_{u,v}\Vert _{L^2(Q)}\\&\quad \le C_2C_r \bar{C}\Vert \xi \Vert _{L^r(Q)}\Vert z_{u,v}\Vert _{L^2(Q)}. \end{aligned}$$

Defining $K_2:=C_2C_r \bar{C}$, (68) follows. The proof for estimate (69) follows by the same argumentation but using (10) and defining the constant $K_s$ accordingly. Finally, we subtract the adjoint states and employ the mean value theorem to find

$$\begin{aligned}&-\frac{d}{dt}(p^\eta _u - p_u)+\mathcal {A}^*(p^\eta _u - p_u) + \frac{\partial f}{\partial y}(x,t,y^\xi _u)(p^\eta _u - p_u) \\&\quad = \frac{\partial ^2 L}{\partial y^2}(x,t,y_\theta )(y^\xi _u-y_u)+\frac{\partial ^2 f}{\partial y^2}(x,t,y_\theta )(y^\xi _u-y_u)p_u+\eta . \end{aligned}$$

The claim follows using (7), (16) and (64), (65) for Remark 4 to estimate

$$\begin{aligned} \Vert p^\eta _u - p_u\Vert _{L^2(Q)}\le (C_2 ^2\bar{C}+M_{{\mathcal {U}}}C_2 ^2{\bar{C}}+C_2 )(\Vert \xi \Vert _{L^2(Q)}+\Vert \eta \Vert _{L^2(Q)}). \end{aligned}$$

$$\square $$

#### Lemma 20

Let $s\in [1,\frac{n+2}{n})\cap [1,2]$. Let $u \in {\mathcal {U}}$ and let $y_u$, $p_u$ be the corresponding state and adjoint state. Further, let $y_u^\zeta$ and $p^\zeta _u$ be solutions to the perturbed state and adjoint equation in (61) for the control *u*. There exist positive constants $C,{\tilde{C}}$, independent of $\zeta \in \Gamma$, such that for $v\in {\mathcal {U}}$, the following estimates hold.

1. For $$m=0$$ in (4):
  71 $$\begin{aligned} \Big \vert \int _Q&\Big (\frac{\partial H}{\partial u}(x,t,y_u,p_u)- \frac{\partial H}{\partial u}(x,t,y^{\zeta }_u,p^{\zeta }_u)\Big )(v-u)\,\textrm{d}x\,\textrm{d}t\Big \vert \nonumber \\&\le C(\Vert \xi \Vert _{L^2(Q)}+\Vert \eta \Vert _{L^2(Q)})\Vert z_{u,u - v}\Vert _{L^2(Q)}\end{aligned}$$
  72 $$\begin{aligned}&\le {\tilde{C}}(\Vert \xi \Vert _{L^2(Q)}+\Vert \eta \Vert _{L^2(Q)})\Vert v-u\Vert _{L^1(Q)}^{\frac{3s-2}{2s}}. \end{aligned}$$
2. For a general $$m\in {\mathbb {R}}$$:
  73 $$\begin{aligned} \Big \vert \int _Q&\Big (\frac{\partial H}{\partial u}(x,t,y_u,p_u)- \frac{\partial H}{\partial u}(x,t,y^{\zeta }_u,p^{\zeta }_u)\Big )(v-u)\,\textrm{d}x\,\textrm{d}t\Big \vert \nonumber \\&\le {\tilde{C}}(\Vert \xi \Vert _{L^r(Q)}+\Vert \eta \Vert _{L^r(Q)})\Vert v-u\Vert _{L^1(Q)}. \end{aligned}$$

#### Proof

We consider the first case, $m=0$. We begin with integrating by parts

$$\begin{aligned}&\Big \vert \int _Q\Big (\frac{\partial H}{\partial u}(x,t,y_u,p_u)-\frac{\partial H}{\partial u}(x,t,y^{\zeta }_u,p^{\zeta }_u)\Big )(v-u)\,\textrm{d}x\,\textrm{d}t\Big \vert \\&\quad \le \Big \vert \int _Q\Big [\frac{\partial L_0}{\partial y}(x,t,y_u)z_{u,u-v}-\frac{\partial L_0}{\partial y}(x,t,y^{\zeta }_u)z_{u,u-v}^{\zeta }\Big ]\,\textrm{d}x\,\textrm{d}t\Big \vert +\Big \vert \int _Q z_{u,u-v}^{\zeta } \eta \,\textrm{d}x\,\textrm{d}t\Big \vert \\&\quad \le \int _Q\Big \vert \frac{\partial L_0}{\partial y}(x,t,y_u)-\frac{\partial L_0}{\partial y}(x,t,y^{\zeta }_u)\Big \vert \Big \vert z_{u,u-v}\Big \vert \,\textrm{d}x\,\textrm{d}t\\&\quad +\int _Q\Big \vert \frac{\partial L_0}{\partial y}(x,t,y^{\zeta }_u)+\eta \Big \vert \Big \vert z_{u,u-v}-z_{u,u-v}^{\zeta }\Big \vert \,\textrm{d}x\,\textrm{d}t\\&\quad +\Big \vert \int _Q \eta z_{u,u-v}\,\textrm{d}x\,\textrm{d}t\Big \vert =I_1+I_2+I_3. \end{aligned}$$

For the first term we use the Hölder inequality, the mean value theorem, (10), (16), Remark 4 and (67) to estimate

$$\begin{aligned} I_1&\le \int _Q\Big \vert \frac{\partial L_0}{\partial y}(x,t,y_u) - \frac{\partial L_0}{\partial y}(x,t,y_{u}^\zeta )\Big \vert \vert z_{u,u - v}\vert \,\textrm{d}x\,\textrm{d}t\\&\le \bar{C}\Vert y^\zeta _{u} - y_{u}\Vert _{L^2(Q)}\Vert z_{u,u - v}\Vert _{L^2(Q)}\\&\le \bar{C}C_2 \Vert \xi \Vert _{L^2(Q)}\Vert z_{u,u - v}\Vert _{L^2(Q)} \\&\le \bar{C}C_2 C_{s'}^{1+\frac{2-s}{2}}(2M_{{\mathcal {U}}})^{\frac{(s'-1)(2-s)}{2s'}} \Vert \xi \Vert _{L^2(Q)}\Vert u-v\Vert _{L^1(Q)}^{1+\frac{s-2}{2s}}. \end{aligned}$$

Here we used that by Theorem 1 and Lemma 10 it holds

$$\begin{aligned} \Vert z_{u,u - v}\Vert _{L^2(Q)}&\le \Vert z_{u,u - v}\Vert _{L^\infty (Q)}^\frac{2-s}{2}\Vert z_{u,u - v}\Vert _{L^s(Q)}^{\frac{s}{2}}\\&\le C_{s'}^{1+\frac{2-s}{2}}(2M_{{\mathcal {U}}})^{\frac{(s'-1)(2-s)}{2s'}}\Vert u-v\Vert _{L^1(Q)}^{\frac{2-s}{2s'}+\frac{s}{2}}, \end{aligned}$$

and noticing that $\frac{2-s}{2s'}+\frac{s}{2}=1-\frac{2-s}{2s}$. The second term is estimated by using (16), Hölder’s inequality, Remark 4 and (68):

$$\begin{aligned} I_2&\le \int _Q\Big \vert \frac{\partial L_0}{\partial y}(x,t,y^\zeta _u)+\eta \Big \vert \Big \vert z^\zeta _{u,u-v} - z_{u,u -v}\Big \vert \,\textrm{d}x\,\textrm{d}t\\&\le 2K_s{\bar{C}} (\Vert \xi \Vert _{L^2(Q)}+\Vert \eta \Vert _{L^2(Q)})\Vert z_{u,u - v}\Vert _{L^2(Q)}\\&\le K(\Vert \xi \Vert _{L^2(Q)}+\Vert \eta \Vert _{L^2(Q)})\Vert u- v\Vert _{L^1(Q)}^{1+\frac{s-2}{2s}}, \end{aligned}$$

where $K:= 2K_s{\bar{C}} C_{s'}^{1+\frac{2-s}{2}}(2M_{{\mathcal {U}}})^{\frac{(s'-1)(2-s)}{2\,s'}}$. For last term we estimate

$$\begin{aligned} I_3 \le \Big \vert \int _Q \eta z_{u,u-v}\,\textrm{d}x\,\textrm{d}t\Big \vert \le \Vert z_{u,u-v}\Vert _{L^2(Q)}\Vert \eta \Vert _{L^2(Q)}. \end{aligned}$$

We prove the second case (73). By applying (8) and arguing as in the proof of (67) and (70) but for *r*, we infer the existence of a positive constant, denoted by ${\tilde{C}}$, such that:

$$\begin{aligned} \Big \vert \int _Q&\Big (\frac{\partial H}{\partial u}(x,t,y_u,p_u)-\frac{\partial H}{\partial u}(x,t,y^{\zeta }_u,p^{\zeta }_u)\Big )(v-u)\,\textrm{d}x\,\textrm{d}t\Big \vert \\&=\Big \vert \int _Q \Big [p_u-p^{\zeta }_u+m(y_u-y^{\zeta }_u)\Big ](v-u)\,\textrm{d}x\,\textrm{d}t\Big \vert \\&\le \Vert p_u-p^{\zeta }_u+m(y_u-y^{\zeta }_u)\Vert _{L^\infty (Q)}\Vert u-{\bar{u}}\Vert _{L^1(Q)}\\&\le {\tilde{C}}(\Vert \xi \Vert _{L^r(Q)}+\Vert \eta \Vert _{L^r(Q)})\Vert v-u\Vert _{L^1(Q)}. \end{aligned}$$

$$\square $$

The main result in the paper follows.

#### Theorem 21

Let condition $(A_0)$ be fulfilled for the reference solution ${\bar{\psi }} = (y_{{\bar{u}}},p_{{\bar{u}}}, {\bar{u}})$ of $0 \in \Phi (\psi )$. Then the mapping $\Phi$ is strongly metrically Hölder subregular at $({\bar{\psi }},0)$. More precisely, for every $\varepsilon \in (0, 1/2]$ there exist positive constants $\alpha _n$ and $\kappa _n$ (with $\alpha _1$ and $\kappa _1$ independent of $\varepsilon$) such that for all $\psi \in {\mathcal {Y}}$ with $\Vert u-{\bar{u}}\Vert _{L^1(Q)} \le \alpha _n$ and $\zeta \in \Gamma$ satisfying $\zeta \in \Phi (\psi )$, the following inequalities are satisfied.

1. In the case $m=0$ in (4):
  74 $$\begin{aligned}&\Vert u-{\bar{u}} \Vert _{L^1(Q)}\le \kappa _n \Big (\Vert \rho \Vert _{L^{\infty }(Q)}+\Vert \xi \Vert _{L^2(Q)}+\Vert \eta \Vert _{L^2(Q)}\Big )^{\theta _0}, \end{aligned}$$
  75 $$\begin{aligned}&\Vert y^{\zeta }_u-y_{{\bar{u}}}\Vert _{L^2(Q)}+\Vert p^{\zeta }_u-p_{{\bar{u}}}\Vert _{L^2(Q)} \le \kappa _n \Big (\Vert \rho \Vert _{L^{\infty }(Q)}+\Vert \xi \Vert _{L^2(Q)}+\Vert \eta \Vert _{L^2(Q)}\Big )^{\theta }, \end{aligned}$$
  where
  76 $$\begin{aligned}&\theta _0 = \theta = 1&\quad \text{ if } \; n = 1,&\qquad \end{aligned}$$
  77 $$\begin{aligned}&\theta _0 = \theta = 1 - \varepsilon&\quad \text{ if } \; n= 2,&\qquad \end{aligned}$$
  78 $$\begin{aligned}&\theta _0 = \frac{10}{11} - \varepsilon , \;\; \theta = \frac{9}{11} - \varepsilon&\quad \text{ if } \; n= 3.&\qquad \end{aligned}$$
2. In the general case $m\in {\mathbb {R}}$:
  79 $$\begin{aligned}&\Vert u-{\bar{u}}\Vert _{L^1(Q)}\le \kappa _n \Big (\Vert \rho \Vert _{L^{\infty }(Q)}+\Vert \xi \Vert _{L^r(Q)}+\Vert \eta \Vert _{L^r(Q)}\Big ), \end{aligned}$$
  80 $$\begin{aligned}&\Vert y^{\zeta }_u-y_{{\bar{u}}}\Vert _{L^2(Q)}+\Vert p^{\zeta }_u-p_{{\bar{u}}}\Vert _{L^2(Q)} \le \kappa _n \Big (\Vert \rho \Vert _{L^{\infty }(Q)}+\Vert \xi \Vert _{L^r(Q)}+\Vert \eta \Vert _{L^r(Q)}\Big )^{\theta _0}. \end{aligned}$$

#### Proof

We begin with the proof for $m=0$. We select $\alpha _1 <{\tilde{\alpha }}_0$ according to Lemma 12. Let $\zeta =(\xi ,\eta ,\rho )\in {\mathcal {Z}}$ and $\psi =(y^{\zeta }_u,p^{\zeta }_u,u)$ with $\Vert u-{\bar{u}}\Vert _{L^1(Q)}\le \alpha _1$ such that $\zeta \in \Phi (\psi )$, i.e.

$$\begin{aligned} \left\{ \begin{array}{cll} \xi &{}=&{}\mathcal Ly^{\zeta }_u+f(\cdot ,\cdot ,y^{\zeta }_u) - u,\\ \eta &{}=&{}{\mathcal {L}}^*p_u^{\zeta }-\frac{\partial H}{\partial y}(\cdot ,y^{\zeta }_u,p_u^{\zeta },u),\\ \rho &{}\in &{} \frac{\partial H}{\partial u}(\cdot ,y^{\zeta }_u,p_u^{\zeta }) + N_{{\mathcal {U}}}(u). \end{array} \right. \end{aligned}$$

Let $y_u$ and $p_u$ denote the solutions to the unperturbed problem with respect to *u*, i.e.

$$\begin{aligned} 0=\mathcal Ly_u+f(\cdot ,\cdot ,y_u)-u\text { and } 0={\mathcal {L}}^*p_u-\frac{\partial H}{\partial y}(\cdot ,y_u,p_u,u). \end{aligned}$$

By Lemma 19, there exist positive constants $$C_2,R_2 $$ independent of $$\psi $$ and $$\zeta $$ such that

81 $$\begin{aligned} \Vert y^{\zeta }_u-y_{u}\Vert _{L^2(Q)}&+\Vert p^{\zeta }_u-p_{u}\Vert _{L^2(Q)}\le (C_2 +R_2 )\Big (\Vert \xi \Vert _{L^2(Q)}+\Vert \eta \Vert _{L^2(Q)}\Big ). \end{aligned}$$

By the definition of the normal cone, $\rho \in \frac{\partial H}{\partial u}(\cdot ,\cdot ,y^{\zeta }_u,p^{\zeta }_u)+ N_{{\mathcal {U}}}(u)$ is equivalent to

$$\begin{aligned} 0\ge \int _Q(\rho -\frac{\partial H}{\partial u}(\cdot ,\cdot ,y^{\zeta }_u,p^{\zeta }_u))(w-u)\ \ \forall w\in {\mathcal {U}}. \end{aligned}$$

We conclude for $w={\bar{u}}$,

82 $$\begin{aligned} 0&\ge \int _Q \frac{\partial H}{\partial u}(\cdot ,\cdot ,y_u,p_u)(u-{\bar{u}})\nonumber \\&\quad +\int _Q(\rho +\frac{\partial H}{\partial u}(\cdot ,\cdot ,y_u,p_u)-\frac{\partial H}{\partial u}(\cdot ,\cdot ,y^{\zeta }_u,p^{\zeta }_u))({\bar{u}}-u)\nonumber \\&\ge J'(u)(u-{\bar{u}})-\Vert \rho \Vert _{L^{\infty }(Q)}\Vert {\bar{u}}-u\Vert _{L^1(Q)}\nonumber \\&\quad -\Big \vert \int _Q(\frac{\partial H}{\partial u}(\cdot ,\cdot ,y_u,p_u)-\frac{\partial H}{\partial u}(\cdot ,\cdot ,y^{\zeta }_u,p^{\zeta }_u))({\bar{u}}-u)\,\textrm{d}x\,\textrm{d}t\Big \vert . \end{aligned}$$

By Lemma 20, we have an estimate on the third term. Since $\Vert u-{\bar{u}}\Vert _{L^1(Q)}<{\tilde{\alpha }}_0$, we estimate by Lemma 12 and Lemma 20

$$\begin{aligned} \Vert u-{\bar{u}}\Vert _{L^1(Q)}^2{\tilde{\gamma }}\le J'(u)(u-{\bar{u}})&\le {\tilde{C}} \Big (\Vert \xi \Vert _{L^2(Q)}+\Vert \eta \Vert _{L^2(Q)}\Big ) \Vert u-{\bar{u}}\Vert _{L^1(Q)}^{1+\frac{s-2}{2s}}\\&+\Vert \rho \Vert _{L^{\infty }(Q)}\Vert {\bar{u}}-u\Vert _{L^1(Q)} \end{aligned}$$

and consequently for an adapted constant, denoted in the same way

$$\begin{aligned} \Vert {\bar{u}}-u\Vert _{L^1(Q)}\le {\tilde{C}}\Big (\Vert \rho \Vert _{L^{\infty }(Q)}+\Vert \xi \Vert _{L^2(Q)}+\Vert \eta \Vert _{L^2(Q)}\Big )^{\frac{2s}{s+2}}. \end{aligned}$$

To estimate the states, we use the estimate for the controls. We notice that $(2-s)/(2s')+s/2=1+(s-2)(2s)$ and obtain

83 $$\begin{aligned} \Vert y_{{\bar{u}}}-y_u\Vert _{L^{2}(Q)}&\le \Vert y_{{\bar{u}}}-y_u \Vert _{L^{\infty }(Q)}^{\frac{2-s}{2}} \Vert y_{{\bar{u}}}-y_u\Vert _{L^s(Q)}^{\frac{s}{2}}\le C_r^{\frac{2-s}{2}}\Vert {\bar{u}}-u \Vert _{L^1(Q)}^{1+\frac{s-2}{2s}}. \end{aligned}$$

Thus, for a constant again denoted by ${\tilde{C}}$ and with

$$\begin{aligned}{} & {} \left( 1+\frac{s-2}{2s}\right) \frac{2s}{s+2}=\frac{3s-2}{2+s},\\{} & {} \Vert y_{{\bar{u}}}-y_u\Vert _{L^{2}(Q)}\le {\tilde{C}} \Big (\Vert \xi \Vert _{L^2(Q)}+\Vert \eta \Vert _{L^2(Q)}+\Vert \rho \Vert _{L^{\infty }(Q)}\Big )^{\frac{3s-2}{2+s}}. \end{aligned}$$

Next, we realize that by Lemma 19 and (4.2)

$$\begin{aligned}&\Vert y_{{\bar{u}}}-y_u^\zeta \Vert _{L^2(Q)}\le \Vert y_{{\bar{u}}}-y_u \Vert _{L^2(Q)}+\Vert y_{u}-y_u^\zeta \Vert _{L^2(Q)}\\&\quad \le \max \left\{ {\tilde{C}},C_2 \right\} \Big (\Vert \xi \Vert _{L^2(Q)}+\Vert \eta \Vert _{L^2(Q)}+\Vert \rho \Vert _{L^{\infty }(Q)}\Big )^{\frac{3s-2}{2+s}}. \end{aligned}$$

Using $\Vert p_{{\bar{u}}}-p_u\Vert _{L^2(Q)}\le C_2 \Vert y_{{\bar{u}}}-y_u\Vert _{L^2(Q)}$ and (70), the same estimate holds for the adjoint state

$$\begin{aligned}&\Vert p_{{\bar{u}}}-p_u^\zeta \Vert _{L^2(Q)}\le \Vert p_{{\bar{u}}}-p_u \Vert _{L^2(Q)}+\Vert p_{u}-p_u^\zeta \Vert _{L^2(Q)}\\&\quad \le (C_2 {\tilde{C}}+R_2 ) \Big (\Vert \xi \Vert _{L^2(Q)}+\Vert \eta \Vert _{L^2(Q)}+\Vert \rho \Vert _{L^{\infty }(Q)}\Big )^{\frac{3s-2}{2+s}}, \end{aligned}$$

subsequently we define $\kappa :=\max \{{\tilde{C}},C_2 \}$. Finally, we consider the case $m\ne 0$. Using estimate 73 in (82) and arguing from that as for the case $m=0$, we infer the existence of a constant ${\tilde{C}}>0$ such that

$$\begin{aligned} \Vert u-{\bar{u}}\Vert _{L^1(Q)}\le {\tilde{C}} \Big ( \Vert \rho \Vert _{L^{\infty }(Q)}+\Vert \xi \Vert _{L^r(Q)}+\Vert \eta \Vert _{L^r(Q)}\Big ). \end{aligned}$$

This implies under (83) the estimate for the states and adjoint-states

$$\begin{aligned}&\Vert y_{{\bar{u}}}-y_u^\zeta \Vert _{L^2(Q)}+\Vert p_{{\bar{u}}}-p_u^\zeta \Vert _{L^2(Q)}\\&\quad \le \max \left\{ {\tilde{C}},C_2 {\tilde{C}}+R_2 \right\} \Big (\Vert \xi \Vert _{L^2(Q)}+\Vert \eta \Vert _{L^2(Q)}+\Vert \rho \Vert _{L^{\infty }(Q)}\Big )^{1+\frac{s-2}{2s}}. \end{aligned}$$

To determine $\theta$ and $\theta _0$ we notice that the functions

$$\begin{aligned} s\rightarrow \frac{s-2}{2s} \text { and } s\rightarrow \frac{3s-2}{2+s} \end{aligned}$$

are monotone. Inserting the value for $(n+2)/2$ for each case $n\in \{1,2,3\}$ completes the proof. $\square$

To obtain results under Assumption 3 for $k\in \{1,2\}$, we need additional restrictions. We either don’t allow perturbations $\rho$ (appearing in the inclusion in (61)) or they need to satisfy

84 $$\begin{aligned} \rho \in D({\mathcal {L}}^*). \end{aligned}$$

#### Theorem 22

Let $m=0$ and let some of the conditions $(A_1),(B_1)$ and $(A_2),(B_2)$ be fulfilled for the reference solution ${\bar{\psi }} = (y_{{\bar{u}}}, p_{{\bar{u}}}, {\bar{u}})$ of $0 \in \Phi (\psi )$. Let, in addition, the set $\Gamma$ of feasible perturbations be restricted to such $\zeta \in \Gamma$ for which the component $\rho$ is either zero or satisfies (84). The numbers $\alpha _n$, $\kappa _n$ and $\varepsilon$ are as in Theorem 21. Then the following statements hold for $n \in \{ 1,2,3\}$:

1. Under Assumption 3, cases $(A_1)$ and $(B_1)$, the estimations
  $$\begin{aligned}&\Vert u-{\bar{u}} \Vert _{L^1(Q)}\le \kappa _n \Big (\Vert {\mathcal {L}}^*\rho \Vert _{L^{2}(Q)}+\Vert \xi \Vert _{L^2(Q)}+\Vert \eta \Vert _{L^2(Q)}\Big ),\\&\Vert y^{\zeta }_u-y_{{\bar{u}}}\Vert _{L^2(Q)}+\Vert p^{\zeta }_u-p_{{\bar{u}}}\Vert _{L^2(Q)} \le \kappa _n \Big (\Vert {\mathcal {L}}^*\rho \Vert _{L^{2}(Q)}+\Vert \xi \Vert _{L^2(Q)}+\Vert \eta \Vert _{L^2(Q)}\Big )^{\theta _0}, \end{aligned}$$
  with $\theta _0$ as in Theorem 21, hold for all $u\in {\mathcal {U}}$ with $\Vert y_u- y_{{\bar{u}}}\Vert _{L^\infty (Q)}<\alpha _n$, in the case of $(A_1)$, or $\Vert u-{\bar{u}} \Vert _{L^1(Q)}<\alpha _n$ in the case ($B_1$), and for all $\zeta \in \Gamma$ satisfying $\zeta \in \Phi (\psi )$.
2. Under Assumption 3, cases $(A_2)$ and $(B_2)$, the estimation
  $$\begin{aligned} \Vert y^{\zeta }_u-y_{{\bar{u}}}\Vert _{L^2(Q)}+\Vert p^{\zeta }_u-p_{{\bar{u}}}\Vert _{L^2(Q)} \le \kappa _n \Big (\Vert {\mathcal {L}}^* \rho \Vert _{L^{2}(Q)}+\Vert \xi \Vert _{L^2(Q)} + \Vert \eta \Vert _{L^2(Q)} \Big ) \end{aligned}$$
  hold for all $u\in {\mathcal {U}}$ with $\Vert y_u-y_{{\bar{u}}}\Vert _{L^\infty (Q)}<\alpha _n$, in the case of $(A_2)$, or $\Vert u-{\bar{u}} \Vert _{L^1(Q)}<\alpha _n$ in the cases ($B_2$), and for all $\zeta \in \Gamma$ satisfying $\zeta \in \Phi (\psi )$.

#### Proof

We first notice that if the perturbation $\rho$ satisfies (84), it holds

$$\begin{aligned} \int _Q \rho (u-{\bar{u}})\,\textrm{d}x\,\textrm{d}t&=\int _Q\left( \left( \frac{d}{dt}+{\mathcal {A}}\right) z_{{\bar{u}},u-{\bar{u}}}+f_y(x,t,y_{{\bar{u}}}\right) z_{{\bar{u}},u-{\bar{u}}})\rho \,\textrm{d}x\,\textrm{d}t\\&=\int _Q\left( \left( -\frac{d}{dt}+{\mathcal {A}}^*\right) \rho +f_y(x,t,y_{{\bar{u}}})\rho \right) z_{{\bar{u}},u-{\bar{u}}}\,\textrm{d}x\,\textrm{d}t. \end{aligned}$$

Thus

$$\begin{aligned}&\Big \vert \int _Q \rho (u-{\bar{u}})\,\textrm{d}x\,\textrm{d}t\Big \vert \\&\quad \le \Vert z_{{\bar{u}},u-{\bar{u}}}\Vert _{L^2(Q)}(\Vert {\mathcal {L}}^* \rho \Vert _{L^2(Q)}+\Vert f_y(x,t,y_{{\bar{u}}})\Vert _{L^\infty (Q)}\Vert \rho \Vert _{L^2(Q)}). \end{aligned}$$

Under Assumption $(A_1)$, we can proceed as in the proof of Theorem 21 using Lemma 12 and (71) in Lemma 20, to infer the existence of positive constants $\alpha ,\kappa$ such that

$$\begin{aligned} \Vert {\bar{u}}-u\Vert _{L^1(Q)}\le \kappa \Big (\Vert {\mathcal {L}}^* \rho \Vert _{L^{2}(Q)}+\Vert \xi \Vert _{L^2(Q)}+\Vert \eta \Vert _{L^2(Q)}\Big ), \end{aligned}$$

and by standard estimates and using (18) the existence of a positive constant *C* such that

$$\begin{aligned}&\Vert y_{{\bar{u}}}-y_u\Vert _{L^2(Q)}+\Vert p_{{\bar{u}}}-p_u\Vert _{L^2(Q)}\le C \Vert y_{{\bar{u}}}-y_u\Vert _{L^2(Q)}\le 2C \Vert z_{u,u-{\bar{u}}}\Vert _{L^2(Q)}\\&\quad \le 2C \kappa ^{\frac{2s}{s+2}}\Big (\Vert {\mathcal {L}}^* \rho \Vert _{L^{2}(Q)}+\Vert \xi \Vert _{L^2(Q)}+\Vert \eta \Vert _{L^2(Q)}\Big )^{\frac{2s}{s+2}}, \end{aligned}$$

for all $u\in {\mathcal {U}}$ with $\Vert y_u-y_{{\bar{u}}}\Vert _{L^\infty (Q)}<\alpha$ or $\Vert u-{\bar{u}} \Vert _{L^1(Q)}<\alpha$ depending on the assumption. From here on, one can proceed as in the proof of Theorem 21 and define the final constant $\kappa >0$ and the exponent $\theta _0$ accordingly. Finally, by similar reasoning, under condition $(A_2)$ with Lemma 12 and Lemma 20, one obtains the existence of a positive constant $\kappa$ such that

$$\begin{aligned}&\Vert y_{{\bar{u}}}-y_u\Vert _{L^2(Q)}+\Vert p_{{\bar{u}}}-p_u\Vert _{L^2(Q)}\le \kappa \Big (\Vert {\mathcal {L}}^* \rho \Vert _{L^{2}(Q)}+\Vert \xi \Vert _{L^2(Q)}+\Vert \eta \Vert _{L^2(Q)}\Big ), \end{aligned}$$

for all $u\in {\mathcal {U}}$ with $\Vert y_u-y_{{\bar{u}}}\Vert _{L^\infty (Q)}<\alpha$ or $\Vert u-{\bar{u}} \Vert _{L^1(Q)}<\alpha$. Again, proceeding as in Theorem 21 and increasing the constant $\kappa$ if needed, proves the claim. $\square$

#### Remark 5

Theorems 21 and 22 concern perturbations which are functions of *x* and *t* only. On the other hand, [15, Theorem ] suggests that SMHSr implies a similar stability property under classes of perturbations that depend (in a non-linear way) on the state and control. This fact will be used and demonstrated in the next section.

## Stability of the optimal solution

In this section we obtain stability results for the optimal solution under non-linear perturbations in the objective functional. Namely, we consider a disturbed problem

85 $$\begin{aligned} {\mathrm{(P}}_{\zeta }{\mathrm{)}}\ \min _{u \in \mathcal {U}} J_\zeta (u) := \int _Q[L(x,t,y(x,t),u(x,t))+ \mu (x,t,y(x,t),u(x,t))]\,\textrm{d}x\,\textrm{d}t, \end{aligned}$$

subject to

86 $$\begin{aligned} \left\{ \begin{array}{l} \frac{dy}{dt}+{\mathcal {A}} y+f(x,t,y) = u + \xi \ \text { in }\ Q,\\ y=0 \ \text { on }\ \Sigma ,\ y(\cdot ,0)=y_0 \text { in } \Omega , \end{array} \right. \end{aligned}$$

where $\zeta := (\xi ,\mu )$ is a perturbation. The corresponding solution will be denoted by $y_u^\zeta$. In contrast with the previous section, the perturbation $\mu$ may be state and control dependent. For this reason, here we change the notation of the set of admissible perturbations to ${\hat{\Gamma }}$. However, Assumption 4 will still be valid for the set ${\hat{\Gamma }}$. The notations $C_{pe}$, $K_y$ and *R* used below have the same meaning as in Sect. 4.2 (see Assumption 4 and the subsequent the paragraph).

In addition to Assumption 4 we require the following that holds through the reminder of the section.

### Assumption 5

For every $\zeta := (\xi ,\mu ) \in {\hat{\Gamma }}$, it holds that $\mu \in L^1(Q \times R)$. For a.e. $(x,t) \in Q$ the function $\mu (x,t,\cdot ,\cdot )$ is of class $C^2$ and is convex with respect to the last argument, *u*. Moreover, the functions $\frac{\partial \mu }{\partial y}$ and $\frac{\partial ^2\mu }{\partial y^2}$ are bounded on $Q \times R$, and the second one is continuous in $(y,u) \in R$, uniformly with respect to $(t,x) \in Q$.

Due to the linearity of (86) and the convexity of the objective functional (85) with respect to *u*, the proof of the next theorem is standard.

### Theorem 23

For perturbations $\zeta \in {\hat{\Gamma }}$ satisfying Assumption 5, the perturbed problem $\mathrm{(P}_{\zeta }\mathrm{)}$ has a global solution.

In the next two theorems, we consider sequences of problems $\{{\mathrm{(P}}_{\zeta _k}\mathrm{)}\}$ with $\zeta _k \in {\hat{\Gamma }}$. The proofs repeat the arguments in [3, Theorem 4.2, Theorem 4.3].

### Theorem 24

Let a sequence $\{\zeta _k \in {\hat{\Gamma }}\}_k$ converge to zero in $L^2(Q) \times L^2(Q \times R)$ and let $u_k$ be a local solution of problem ($P_{\zeta _k}$), $k = 1, \, 2, \ldots$. Then any control ${\bar{u}}$ that is a weak* limit in $L^\infty (Q)$ of this sequence is a weak local minimizer in problem (P), and for the corresponding solutions it holds that $y_{u_k} \rightarrow y_{{\bar{u}}}$ in $L^2(0,T\text {; }H^1_0(\Omega ))\cap L^\infty (Q)$.

### Theorem 25

Let $\{\zeta _k\}_k$ be as in Theorem 24. Let ${\bar{u}}$ be a strict strong local minimizer of (P). Then there exists a sequence of strong local minimizers $\{u_k\}$ of problems ($P_{\zeta _k}$) such that $u_k {\mathop {\rightharpoonup }\limits ^{*}} {\bar{u}}$ in $L^\infty (Q)$ and $y_{u_k}$ converges strongly in $L^2(0,T; H^1_0(\Omega ))\cap L^\infty (Q)$.

The next theorem is central in this section.

### Theorem 26

Let condition $(A_0)$ be fulfilled for the reference solution ${\bar{\psi }} = (y_{{\bar{u}}},p_{{\bar{u}}}, {\bar{u}})$ of $0 \in \Phi (\psi )$. Then there exist positive numbers *C* and $\alpha$ for which the following is fulfilled. For all $\psi \in {\mathcal {Y}}$ with $\Vert u-{\bar{u}}\Vert _{L^1(Q)} \le \alpha$ and $\zeta \in {\hat{\Gamma }}$ satisfying $\zeta \in \Phi (\psi )$ it holds:

1. If $$m=0$$ in (4):
  $$\begin{aligned} \Vert u-{\bar{u}}\Vert _{L^1(Q)}&\le C\Big [ \Vert \xi \Vert _{L^2(Q)} +\Big \Vert \frac{d}{dy}\mu \Big \Vert _{L^\infty (R;L^2(Q))} \\&\quad + \Big \Vert \frac{d}{du}\mu \Big \Vert _{L^{\infty }(Q \times R)}\Big ]^{\theta _0},\\ \Vert y_{u}^\zeta -y_{{\bar{u}}} \Vert _{L^2(Q)}&\le C\Big [ \Vert \xi \Vert _{L^2(Q)} +\Big \Vert \frac{d}{dy}\mu \Big \Vert _{L^\infty (R;L^2(Q))} \\&\quad + \Big \Vert \frac{d}{du}\mu \Big \Vert _{L^{\infty }(Q \times R)}\Big ]^{\theta }. \end{aligned}$$
2. For $$m\in {\mathbb {R}}$$:
  $$\begin{aligned} \Vert u-{\bar{u}}\Vert _{L^1(Q)}&\le C\Big [ \Vert \xi \Vert _{L^r(Q)} +\Big \Vert \frac{d}{dy}\mu \Big \Vert _{L^\infty (R;L^r(Q))} \\&\quad + \Big \Vert \frac{d}{du}\mu \Big \Vert _{L^{\infty }(Q \times R)}\Big ], \end{aligned}$$
  $$\begin{aligned} \Vert y_{u}^\zeta -y_{{\bar{u}}} \Vert _{L^2(Q)}&\le C\Big [ \Vert \xi \Vert _{L^r(Q)} +\Big \Vert \frac{d}{dy}\mu \Big \Vert _{L^\infty (R;L^r(Q))} \\&\quad + \Big \Vert \frac{d}{du}\mu \Big \Vert _{L^{\infty }(Q \times R)}\Big ]^{\theta _0}. \end{aligned}$$

Here $\theta _0$ and $\theta$ are defined as in Theorem 21.

### Proof

The reference solution $(y_{{\bar{u}}}, {\bar{u}})$ satisfies, together with the corresponding adjoint variable, the relations (58). Similarly, $(y_{u}^\zeta , u)$ satisfies, together with the corresponding $p_{u}^\zeta$ the perturbed optimality system (61) with the left-hand side given by the triple

87 $$\begin{aligned} \left( \begin{array}{cll} \xi (\cdot ) \\ \frac{d}{dy}(\mu (\cdot ,y_{u}^\zeta (\cdot ), u(\cdot )) \\ \frac{d}{du}(\mu (\cdot ,y_{u}^\zeta (\cdot ), u(\cdot )). \end{array} \right) \end{aligned}$$

Since it is assumed that $\Vert u - {\bar{u}} \Vert _{L^1(Q)} \le \alpha$ we may apply Theorem 21 (here we choose the same $\alpha$ as in this theorem) to prove the inequalities in the theorem. $\square$

The proof of theorems 27 and 28 follows in the same spirit but using Theorem 22 instead of Theorem 21. We make an additional assumption for the perturbation $\mu$ in the objective functional, namely, that $\rho := \frac{d}{du}(\mu (\cdot ,y_{u}^\zeta (\cdot ), u(\cdot ))$ satisfies (84), i.e.

88 $$\begin{aligned} \frac{d}{du}(\mu (\cdot ,y_{u}^\zeta (\cdot ), u(\cdot ))\in D({\mathcal {L}}^*). \end{aligned}$$

For an explanation of the condition (88), we refer to the proof of Theorem 22.

### Theorem 27

Let $m=0$ and let condition (A$_1$) be fulfilled for the reference solution ${\bar{\psi }} = (y_{{\bar{u}}},p_{{\bar{u}}}, {\bar{u}})$ of $0 \in \Phi (\psi )$. Then there exist positive numbers $\alpha$ and *C* for which the following is fulfilled. For all $\psi \in {\mathcal {Y}}$ with $\Vert y_{u}-y_{{\bar{u}}}\Vert _{L^\infty (Q)} \le \alpha$ and $\zeta \in {\hat{\Gamma }}$ satisfying $\zeta \in \Phi (\psi )$ and (88) the following estimates hold:

$$\begin{aligned}&\Vert u-{\bar{u}}\Vert _{L^1(Q)}\\&\quad \le C\Big (\Vert {\mathcal {L}}^* \frac{d}{du}(\mu (\cdot ,y_{u}^\zeta (\cdot ), u(\cdot )) \Vert _{L^{2}(Q)}+\Vert \xi \Vert _{L^2(Q)}+\Big \Vert \frac{d}{dy}\mu \Big \Vert _{L^\infty (R;L^2(Q))}\Big ) \end{aligned}$$

and

$$\begin{aligned}&\Vert y_{u}^\zeta -y_{{\bar{u}}}\Vert _{L^2(Q)}\\&\quad \le C\Big (\Vert {\mathcal {L}}^* \frac{d}{du}(\mu (\cdot ,y_{u}^\zeta (\cdot ), u(\cdot ))\Vert _{L^{2}(Q)}+\Vert \xi \Vert _{L^2(Q)}+\Big \Vert \frac{d}{dy}\mu \Big \Vert _{L^\infty (R;L^2(Q))}\Big )^{\theta _0}, \end{aligned}$$

where $\theta _0$ is defined in Theorem 21.

### Theorem 28

Let $m=0$ and let condition (A$_2$) be fulfilled for the reference solution ${\bar{\psi }} = (y_{{\bar{u}}},p_{{\bar{u}}}, {\bar{u}})$ of $0 \in \Phi (\psi )$. Then there exist positive numbers *C* and $\alpha$ for which the following is fulfilled. For all $\psi \in {\mathcal {Y}}$ with $\Vert y_{u}-y_{{\bar{u}}}\Vert _{L^\infty (Q)} \le \alpha$ and $\zeta \in {\hat{\Gamma }}$ satisfying $\zeta \in \Phi (\psi )$ and (88) the following estimate holds.

$$\begin{aligned}&\Vert y_{u}^\zeta -y_{{\bar{u}}}\Vert _{L^2(Q)}\\&\quad \le C\Big (\Vert {\mathcal {L}}^* \frac{d}{du}(\mu (\cdot ,y_{u}^\zeta (\cdot ), u(\cdot )) \Vert _{L^{2}(Q)}+\Vert \xi \Vert _{L^2(Q)}+\Big \Vert \frac{d}{dy}\mu \Big \Vert _{L^\infty (R;L^2(Q))}\Big ). \end{aligned}$$

### Remark 6

The constraint that $u_\zeta$ needs to be close to the reference solution ${\bar{u}}$ in the theorems above is not a big restriction. This is clear, since Assumption 3 implies that ${\bar{u}}$ satisfies (38). Hence, ${\bar{u}}$ is a strict strong local minimizer of (P) and, consequently, Theorem 25 ensures the existence of a family $\{u_{\zeta _k}\}$, ${\zeta _k \in {\hat{\Gamma }}}$, of strong local minimizers of problems ${\mathrm{(P}}_{\zeta }\mathrm{)}$ satisfying the conditions of Theorem 21 or 22.

## Examples

Here, we present three examples that show particular applications in which different assumptions are involved.

### Example 1

(Tikhonov regularization) We consider the optimal control problem

$$\begin{aligned} {\mathrm{(P}}_{\lambda }{\mathrm{)}}\ \min _{u \in \mathcal {U}} J_\lambda (u):= \int _Q L(x,t,y(x,t),u(x,t)) + \frac{\lambda }{2}\int _Q u(x,t)^2\,\textrm{d}x\,\textrm{d}t, \end{aligned}$$

subject to (2) and (3). As before, ${\bar{u}}$ denotes a strict strong solution of problem (P)$\equiv \mathrm{(P}_{0}\mathrm{)}$. We assume that ${\bar{u}}$ satisfies condition $(A_0)$. From Theorem 25 we know that for every sequence $\lambda _k>0$ converging to zero there exists a sequence of strong local minimizer $\{ u_{\lambda _k}\}_{k=1}^\infty$ such that $u_{\lambda _k}\rightarrow {\bar{u}}$ in $L^1(Q)$ for $k\rightarrow \infty$, thus for a sufficiently large $k_0$ we have that for all $k>k_0$ and a positive constant *C*

$$\begin{aligned}&\Vert y_{{\bar{u}}}-y_{u_{\lambda _k}}\Vert _{L^2(Q)}+\Vert p_{{\bar{u}}}-p_{u_{\lambda _k}} \Vert _{L^2(Q)}\le C\Big (\lambda _k \Big )^{\theta },\\&\quad \Vert {\bar{u}}-u_{\lambda _k}\Vert _{L^1(Q)}\le C\lambda _k, \end{aligned}$$

where $\theta$ is defined in Theorem 21.

### Example 2

(Negative curvature) We consider an optimal control problem, that has negative curvature. The parabolic equation has the form

89 $$\begin{aligned} \left\{ \begin{array}{lll} \frac{dy}{dt}+{\mathcal {A}} y+\exp (y)&{} =u\ &{}\text { in }\ Q,\\ y=0 \text { on } \Sigma ,\quad y(\cdot ,0)&{} = 0\ {} &{}\text { on } \Omega .\\ \end{array} \right. \end{aligned}$$

Let $0 < g \in L^2(Q)$ be a function satisfying the structural assumption, i.e. *g* satisfies (34) in place of $\frac{\partial {\bar{H}}}{\partial u}$. We consider the optimal control problem

$$\begin{aligned} \min _{u\in {\mathcal {U}}} \Big \{ J(u):=\int _Q (y_u +gu) \,\textrm{d}x\,\textrm{d}t\Big \} \end{aligned}$$

subject to (89) and with control constraints

90 $$\begin{aligned} \mathcal {U}:= \{u \in L^\infty (Q) \vert \ 0\le u_a \le u \le u_b\ \text { for a.a. } (x,t) \in Q\}. \end{aligned}$$

By the weak maximum principle $y_{u_a}-y_u\le 0$ for all $u\in {\mathcal {U}}$ and ${\bar{u}}:=u_a$ constitutes an optimal solution. Further, by the weak maximum principle, the adjoint-state $p_{{\bar{u}}}$ and the linearized states $z_{{\bar{u}},u-{\bar{u}}}$ for all $u \in {\mathcal {U}}$, are non-negative. Moreover, we have

$$\begin{aligned} J'({\bar{u}})(u-{\bar{u}}) = \int _Q(p_{{\bar{u}}}+g)(u - {\bar{u}})\,\textrm{d}x\,\textrm{d}t\ge 0, \end{aligned}$$

$$\begin{aligned} J''({\bar{u}})(u-{\bar{u}})^2=\int _Q w_{{\bar{u}},u-{\bar{u}}}\,\textrm{d}x\,\textrm{d}t=\int _Q -p_{{\bar{u}}} \exp ({\bar{y}}) z_{{\bar{u}},u-{\bar{u}}}^2\,\textrm{d}x\,\textrm{d}t< 0, \end{aligned}$$

for all $u \in \mathcal {U}/\bar{u}$. Since *g* satisfies the structural assumption, there exists a constant $C>0$ such that

$$\begin{aligned} \int _Q g(u-{\bar{u}})\,\textrm{d}x\,\textrm{d}t\ge C\Vert u-{\bar{u}}\Vert _{L^1(Q)}^2\ \ \forall u\in {\mathcal {U}}. \end{aligned}$$

On the other hand, integrating by parts we obtain

91 $$\begin{aligned} \int _Q p_{{\bar{u}}}(u-{\bar{u}})\,\textrm{d}x\,\textrm{d}t=\int _Q z_{{\bar{u}}, u-{\bar{u}}}\,\textrm{d}x\,\textrm{d}t. \end{aligned}$$

If for $u\in {\mathcal {U}}$, $\Vert y_u-y_{{\bar{u}}}\Vert _{L^\infty (Q)}$ is sufficiently small such that

$$\begin{aligned} \frac{1}{2\Vert p_{{\bar{u}}} \exp (y_{{\bar{u}}})\Vert _{L^\infty (Q)}}>\Vert z_{{\bar{u}},u-{\bar{u}}}\Vert _{L^\infty (Q)}, \end{aligned}$$

we can absorb the term $J''({\bar{u}})(u-{\bar{u}})^2$ by estimating

92 $$\begin{aligned} J'({\bar{u}})(u-{\bar{u}})+J''({\bar{u}})(u-{\bar{u}})^2&= \int _Q z_{{\bar{u}}, u-{\bar{u}}}(1- p_{{\bar{u}}} \exp (y_{{\bar{u}}}) z_{{\bar{u}},u-{\bar{u}}})\,\textrm{d}x\,\textrm{d}t\end{aligned}$$

93 $$\begin{aligned}&\ge \frac{1}{2}\int _Q z_{{\bar{u}}, u-{\bar{u}}}\,\textrm{d}x\,\textrm{d}t\ge \frac{K}{2}\Vert z_{{\bar{u}}, u-{\bar{u}}}\Vert _{L^2(Q)}^2, \end{aligned}$$

where the last inequality is a consequence of the boundedness of ${\mathcal {U}}\subset L^\infty (Q)$ that implies the existence of a positive constant *K* such that

$$\begin{aligned} \Vert z_{{\bar{u}}, u-{\bar{u}}}\Vert _{L^1(Q)}\ge K\Vert z_{{\bar{u}}, u-{\bar{u}}}\Vert _{L^2(Q)}^2 \end{aligned}$$

for all $u\in {\mathcal {U}}$. Altogether, we find

$$\begin{aligned} J'({\bar{u}})(u-{\bar{u}})+J''({\bar{u}})(u-{\bar{u}})^2&\ge C \Vert u-{\bar{u}}\Vert _{L^1(Q)}^2 + \frac{K}{2} \Vert z_{{\bar{u}}, u-{\bar{u}}}\Vert _{L^2(Q)}^2 \\&\ge \sqrt{\frac{CK}{2}} \Vert u-{\bar{u}}\Vert _{L^1(Q)}\Vert z_{{\bar{u}}, u-{\bar{u}}}\Vert _{L^2(Q)} \ \ \forall u\in {\mathcal {U}}. \end{aligned}$$

Thus, condition $(A_1)$ is fulfilled and we can apply Theorem 22 to obtain a stability result.

### Example 3

(State stability) We will discuss $(A_2)$ for an optimal control problem with tracking type objective functional where the control does not appear explicitly in the objective functional:

$$\begin{aligned} \min _{u\in {\mathcal {U}}} \Big \{ J(u):=\frac{1}{2}\int _Q (y_u -y_d)^2 \,\textrm{d}x\,\textrm{d}t\Big \} \end{aligned}$$

subject to (3) and equation (89) and a given function $y_d\in L^r(Q)$. As perturbations we consider functions $\zeta :=(\xi ,\eta , \rho ) \in L^r(Q)\times L^r(Q)\times D({\mathcal {L}}^*)$. Denote by ${\bar{\psi }} = (y_{{\bar{u}}},p_{{\bar{u}}}, {\bar{u}})$ the reference solution of $0 \in \Phi (\psi )$ satisfying $(A_2)$ and consider the perturbed problem

$$\begin{aligned} \min _{u \in \mathcal {U}} \Big \{J(u):= \frac{1}{2}\int _Q (y(x,t)-y_d(x,t))^2\,\textrm{d}x\,\textrm{d}t+\int _Q\eta y\,\textrm{d}x\,\textrm{d}t+\int _Q\rho u\,\textrm{d}x\,\textrm{d}t\Big \}, \end{aligned}$$

subject to (3) and

$$\begin{aligned} \left\{ \begin{array}{lll} \frac{dy}{dt}+{\mathcal {A}} y+\exp (y)&{} =u+\xi \ &{}\text { in }\ Q,\\ y=0 \text { on } \Sigma ,\quad y(\cdot ,0)&{} = 0\ {} &{}\text { on } \Omega .\\ \end{array} \right. \end{aligned}$$

Condition $(A_2)$ implies that ${\bar{u}}$ is a strong local minimizer of the unperturbed problem ($\zeta =0$), thus it holds

$$\begin{aligned} J'({\bar{u}})(u-{\bar{u}})&=\int _Q(y_{{\bar{u}}}(x,t)-y_d(x,t))z_{{\bar{u}}, u-{\bar{u}}}\ \text {dxdt}\ge 0 \quad \forall u \in \mathcal {U},\\ J''({\bar{u}})(u-{\bar{u}})&=\int _Q(y_{{\bar{u}}}(x,t)-y_d(x,t))w_{{\bar{u}}, u-{\bar{u}}}+z_{{\bar{u}}, u-{\bar{u}}}^2 \ \text {dxdt}\\&=\int _Q(1-p_{{\bar{u}}} \,exp(y_{{\bar{u}}} ))z_{{\bar{u}}, u-{\bar{u}}}^2 \ \text {dxdt} \quad \forall u \in \mathcal {U}, \end{aligned}$$

where $$p_{{\bar{u}}}$$ solves

$$\begin{aligned} \left\{ \begin{array}{lll} -\frac{d p_{{\bar{u}}}}{dt}+{\mathcal {A}}^* p_{{\bar{u}}} + \exp (y_{{\bar{u}}}) p_{{\bar{u}}} = y_{{\bar{u}}}-y_d&{} \text { in } Q,\\ p_{{\bar{u}}} = 0\text { on } \Sigma , \ p_{{\bar{u}}}(\cdot ,T)=0\ {} &{}\text { on } \Omega .\\ \end{array}\right. \end{aligned}$$

If the optimal state tracks $y_d$ such that $\Vert y_{{\bar{u}}}-y_d\Vert _{L^r(Q)}<\frac{1}{C_r \Vert \exp (y_{{\bar{u}}})\Vert _{L^\infty (Q)}}$ we find that $(A_2)$ holds. From Theorem 27 we obtain the existence of positive constants $\alpha$ and $\kappa$ such that

$$\begin{aligned} \Vert y_{{\bar{u}}}-y_{u}^\zeta \Vert _{L^2(Q)}+\Vert p_{{\bar{u}}}-p_{u}^\zeta \Vert _{L^2(Q)} \le \kappa \Big ( \Vert \xi \Vert _{L^2(Q)} + \Vert \eta \Vert _{L^2(Q)} + \Vert {\mathcal {L}}^*\rho \Vert _{L^{2}(Q)} \Big ), \end{aligned}$$

for all $(y^{\zeta }_u,p^{\zeta }_u,u)=\psi \in \Gamma$ with $\Vert y_{u}- y_{{\bar{u}}} \Vert _{L^\infty (Q)} \le \alpha$ and $\zeta \in \Gamma$ satisfying (84) and $\zeta \in \Phi (\psi )$.

## Data Availability

Our work is concerned with a theoretical mathematical approach, thus we do not analyze or generate any datasets.

## Appendix A

### Lemma 29

Suppose $r >1+ \frac{n}{2}$ and $s\in [1,\frac{n+2}{n})\cap [1,2]$. The following statement is fulfilled for all $u,{\bar{u}} \in \mathcal {U}$. There exist positive constants $K_r$, $M_s$ and $N_{r,s}$ depending on *s* and *r* such that

A1 $$\begin{aligned}&\Vert y_u - y_{{\bar{u}}} - z_{{\bar{u}},u - {\bar{u}}}\Vert _{L^\infty (Q)}\le K_r\Vert y_u - y_{{\bar{u}}}\Vert ^2_{L^{2r}(Q)}, \end{aligned}$$

A2 $$\begin{aligned}&\Vert y_u - y_{{\bar{u}}} - z_{{\bar{u}},u - {\bar{u}}}\Vert _{L^{s}(Q)} \le M_s \Vert y_u-y_{{\bar{u}}}\Vert ^{2-s}_{L^\infty (Q)}\Vert y_u-y_{{\bar{u}}}\Vert ^s_{L^{s}(Q)}, \end{aligned}$$

A3 $$\begin{aligned}&\Vert y_u - y_{{\bar{u}}} - z_{{\bar{u}},u - {\bar{u}}}\Vert _{L^{2}(Q)} \le N_{r,s}\Vert y_u-y_{{\bar{u}}}\Vert ^{2-\frac{s^2}{2}}_{L^\infty (Q)}\Vert y_u-y_{{\bar{u}}}\Vert _{L^{s}(Q)}^{\frac{s^2}{2}}. \end{aligned}$$

### Proof

Let us denote $\phi := y_u - y_{{\bar{u}}} - z_{{\bar{u}},u - {\bar{u}}} \in W(0,T) \cap L^\infty (Q)$. From the equations satisfied by the three functions and by the mean value theorem $\phi$ satisfies

$$\begin{aligned} \frac{d \phi }{dt}+\mathcal {A}\phi + \frac{\partial f}{\partial y}(x,t,y_{{\bar{u}}})\phi = \Big [\frac{\partial f}{\partial y}(x,t,y_{{\bar{u}}}) - \frac{\partial f}{\partial y}(x,t,y_\theta )\Big ](y_u - y_{{\bar{u}}}), \end{aligned}$$

where $y_\theta (x,t) = y_{{\bar{u}}}(x,t) + \theta (x,t)(y_u(x,t) - y_{{\bar{u}}}(x,t))$ with $\theta : Q \longrightarrow [0,1]$ measurable. Applying again the mean value theorem we obtain

$$\begin{aligned} \frac{d \phi }{dt}+\mathcal {A}\phi + \frac{\partial f}{\partial y}(x,t,y_{{\bar{u}}})\phi = \theta \frac{\partial ^2f}{\partial y^2}(x,t,y_\vartheta )(y_u - y_{{\bar{u}}})^2 \end{aligned}$$

with $y_\vartheta (x,t) = y_{{\bar{u}}}(x,t) + \vartheta (x,t)(y_\theta (x,t) - y_{{\bar{u}}}(x,t))$ and $\vartheta :Q \longrightarrow [0,1]$ measurable. By Theorem 1 and Remark 4 we infer the existence of constants $C_r, {\bar{C}}$ independent of $u, {\bar{u}} \in \mathcal {U}$ and $\frac{\partial f}{\partial y}(x,t,y_{{\bar{u}}})$ such that

$$\begin{aligned} \Vert \phi \Vert _{L^\infty (Q)} \le C_r\bar{C}\Vert (y_{u}-y_{{\bar{u}}})^2\Vert _{L^r(Q)} = C_r\bar{C}\Vert y_{u}-y_{{\bar{u}}}\Vert ^2_{L^{2r}(Q)}, \end{aligned}$$

which proves (A1) with $K_r:= C_r\bar{C}$. To prove (A2), we use Lemma 2, Remark 4 and (16) to obtain that

$$\begin{aligned} \Vert \phi \Vert _{L^{s}(Q)}&\le C_{s'}\bar{C}\Vert (y_u-y_{{\bar{u}}})^2\Vert _{L^1(Q)}\\&\le C_{s'}\bar{C}\Vert y_u-y_{{\bar{u}}}\Vert ^{2-s}_{L^\infty (Q)}\Vert y_u-y_{{\bar{u}}}\Vert ^s_{L^s(Q)}. \end{aligned}$$

Taking $M_s:= C_{s'}\bar{C}$, (A2) follows. The inequality, (A3), follows from (A2) and (A1) of Lemma 29 by estimating

$$\begin{aligned} \begin{aligned} \Vert \phi \Vert _{L^{2}(Q)}&\le \Vert \phi \Vert _{L^\infty (Q)}^{\frac{2-s}{2}}\Vert \phi \Vert _{L^s(Q)}^{\frac{s}{2}}\\&\le K_{r}^{\frac{2-s}{2}}\Vert y_u-y_{{\bar{u}}}\Vert _{L^{2r}(Q)}^{\frac{2(2-s)}{2}}\bigg [M_s^{\frac{s}{2}}\Vert y_u-y_{{\bar{u}}} \Vert _{L^\infty (Q)}^{\frac{(2-s)s}{2}}\Vert y_u-y_{{\bar{u}}}\Vert _{L^s(Q)}^{\frac{s^2}{2}}\bigg ]\\&\le K_{r}^{\frac{(2-s)}{2}}M_s^{\frac{s}{2}}\vert Q\vert ^{\frac{2-s}{2r}}\Vert y_u-y_{{\bar{u}}} \Vert _{L^\infty (Q)}^{2-s+\frac{(2-s)s}{2}}\Vert y_u-y_{{\bar{u}}}\Vert _{L^s(Q)}^{\frac{s^2}{2}}. \end{aligned} \end{aligned}$$

Defining $N_{r,s}:=K_{r}^{\frac{(2-s)}{2}}M_s^{\frac{s}{2}}\vert Q\vert ^{\frac{2-s}{2r}}$ and noticing that

$$\begin{aligned} 2-s+\frac{(2-s)s}{2}=2-\frac{s^2}{2}, \end{aligned}$$

proves the claim. $\square$

### Proof of Lemma 5

We prove (17) by applying Theorem 1 to $\psi :=z_{{\bar{u}},v}-z_{u_\theta ,v}$, that solves

A4 $$\begin{aligned} \frac{d \psi }{dt}+\mathcal {A}\psi + \frac{\partial f}{\partial y}(x,t,y_{{\bar{u}}})\psi&= \Big [ \frac{\partial f}{\partial y}(x,t,y_{u_\theta })-\frac{\partial f}{\partial y}(x,t,y_{{\bar{u}}})\Big ]z_{u_\theta ,v}\nonumber \\&=\theta \frac{\partial ^2f}{\partial y^2}(x,t,y_\vartheta )(y_{{\bar{u}}} - y_{u_\theta })z_{u_\theta ,v}. \end{aligned}$$

To prove (18), we use (A3) with $s=\sqrt{2}$ to estimate

$$\begin{aligned}&\Vert y_u-y_{{\bar{u}}} \Vert _{L^2(Q)}\le \Vert \phi \Vert _{L^2(Q)}+\Vert z_{\bar{u}, u-{\bar{u}}}\Vert _{L^2(Q)}\\&\quad \le N_{r,\sqrt{2}} \Vert y_u-y_{{\bar{u}}}\Vert _{L^\infty (Q)}\Vert y_u-y_{{\bar{u}}}\Vert _{L^{\sqrt{2}}(Q)}+\Vert z_{\bar{u}, u-{\bar{u}}}\Vert _{L^2(Q)}. \end{aligned}$$

Using fact that by the Hölder inequality $\Vert y_u-y_{{\bar{u}}} \Vert _{L^{\sqrt{2}}(Q)}\le \vert Q\vert ^{\frac{1}{\sqrt{2}}-\frac{1}{2}}\Vert y_u-y_{{\bar{u}}}\Vert _{L^2(Q)}$, the claim follows. For the other direction, we select again $s=\sqrt{2}$ in (A3) and find

$$\begin{aligned} \Vert z_{\bar{u}, u-{\bar{u}}}\Vert _{L^2(Q)}&\le \Vert \phi \Vert _{L^2(Q)}+\Vert y_u-y_{{\bar{u}}}\Vert _{L^2(Q)}\\&\le N_{r,\sqrt{2}} \Vert y_u-y_{{\bar{u}}}\Vert _{L^\infty (Q)}\Vert y_u-y_{{\bar{u}}}\Vert _{L^{\sqrt{2}}(Q)}+\Vert y_u-y_{{\bar{u}}}\Vert _{L^2(Q)}\\&\le \bigg ( N_{r,\sqrt{2}}\vert Q\vert ^{\frac{1}{\sqrt{2}}-\frac{1}{2}}\Vert y_u-y_{{\bar{u}}}\Vert _{L^\infty (Q)}+1\bigg ) \Vert y_u-y_{{\bar{u}}} \Vert _{L^2(Q)}. \end{aligned}$$

Finally, for (19) we use (17) and estimate

$$\begin{aligned} \begin{aligned} \Vert z_{{\bar{u}},v}\Vert _{L^2(Q)}&\le \Vert z_{{\bar{u}},v}-z_{u,v} \Vert _{L^2(Q)}+\Vert z_{ u,v}\Vert _{L^2(Q)}\\&\le K_2\root 2 \of {\vert Q\vert }\Vert y_u - y_{{\bar{u}}}\Vert _{L^\infty (Q)}\Vert z_{{\bar{u}},v}\Vert _{L^2(Q)}+\Vert z_{ u,v}\Vert _{L^2(Q)}. \end{aligned} \end{aligned}$$

Choosing $\varepsilon = [2K_2\root 2 \of {\vert Q\vert }]^{-1}$ proves the first part. The second inequality follows in a similar way. The estimates with respect to the $\Vert \cdot \Vert _{L^\infty (Q)}$-norm follow by similar reasoning, using (A1). $\square$

### Proof of Proposition 8

Let us prove first the implication $(A_k) {\Rightarrow } (B_k)$ for any $k\in \{0,1,2\}$. Given $u \in \mathcal {U}$, by the mean value theorem

$$\begin{aligned} \frac{d(y_u- y_{{\bar{u}}})}{dt}+\mathcal {A}(y_u - y_{{\bar{u}}}) + \frac{\partial f}{\partial y}(x,y_{{\bar{u}}} + \theta (y_u - y_{{\bar{u}}}))(y_u - y_{{\bar{u}}}) = u - {\bar{u}}. \end{aligned}$$

Using (8) in Theorem 1 we obtain that

$$\begin{aligned} \Vert y_u -y_{{\bar{u}}}\Vert _{L^\infty (Q)} \le C_r\Vert u - {\bar{u}}\Vert _{L^r(Q)} \le C_r(2M_{{\mathcal {U}}})^{\frac{r-1}{r}}\Vert u - {\bar{u}}\Vert ^{\frac{1}{r}}_{L^1(Q)}. \end{aligned}$$

Then, by ${\tilde{\alpha }}_k:= \frac{\alpha _k ^r}{C^r_r(2M_{{\mathcal {U}}})^{r-1}}$, we obtain that $(A_k)$ implies $(B_k)$ with $\gamma _k = {\tilde{\gamma }}_k$.

To prove the converse implication, $(B_k) {\Rightarrow } (A_k)$, we assume that ($B_k$) holds, but ($A_k$) fails. Then for every integer $l \ge 1$ there exists an element $u_l \in \mathcal {U}$ such that

A5 $$\begin{aligned} \begin{aligned} J'({\bar{u}})(u_l - {\bar{u}})&+ J''({\bar{u}})(u_l - {\bar{u}})^2\\&< \frac{1}{l}\Vert u_l - {\bar{u}}\Vert ^{2-k}_{L^1(Q)}\Vert z_{{\bar{u}},u_l - {\bar{u}}}\Vert ^{k}_{L^2(Q)}\ \text { and } \ \Vert y_{u_l} - y_{{\bar{u}}}\Vert _{L^\infty (Q)} < \frac{1}{l}. \end{aligned} \end{aligned}$$

Since $\{u_l\}_{l = 1}^\infty \subset \mathcal {U}$ is bounded in $L^\infty (Q)$, we can extract a subsequence, denoted in the same way, such that $u_l {\mathop {\rightharpoonup }\limits ^{*}} u$ in $L^\infty (Q)$. On one side, (A5) implies that $y_{u_l} \rightarrow y_{{\bar{u}}}$ in $L^\infty (Q)$. On the other side, $u_l {\mathop {\rightharpoonup }\limits ^{*}} u$ in $L^\infty (Q)$ implies weak convergence in $L^r(Q)$. From (13), the convergence $y_{u_l} \rightarrow y_u$ in $L^\infty (Q)$ follows. Then, $y_u = y_{{\bar{u}}}$ and, consequently, $u = {\bar{u}}$ holds. But condition ($B_0$) implies that ${\bar{u}}$ is bang-bang, and hence the weak convergence $u_l {\mathop {\rightharpoonup }\limits ^{*}} {\bar{u}}$ in $L^\infty (Q)$ yields the strong convergence $u_l \rightarrow {\bar{u}}$ in $L^1(Q)$; see [17, Proposition 4.1 and Lemma 4.2]. Then, for $k=0$, (A5) contradicts $(B_0)$. The same argument holds for $(B_1)$ and $(B_2)$ under the additional condition that ${\bar{u}}$ is bang-bang and noticing that $\Vert z_{{\bar{u}},u_l-{\bar{u}}}\Vert _{L^\infty (Q)} \le 3/2\Vert y_{u_l}-y_{{\bar{u}}}\Vert _{L^\infty (Q)}$ by Lemma 5. $\square$

A proof of the following lemma can be found in [8, Lemma 3.5].

### Lemma 30

Given ${\bar{u}} \in \mathcal {U}$ with associated state $y_{{\bar{u}}}$. Then, the following estimate holds

A6 $$\begin{aligned} \Vert y_{{\bar{u}} + \theta (u - {\bar{u}})} - y_{{\bar{u}}}\Vert _{L^\infty (Q)} \le B\Vert y_u -y_{{\bar{u}}}\Vert _{L^\infty (Q)} \quad \forall \theta \in [0,1]\ \text { and }\ \forall u \in \mathcal {U}, \end{aligned}$$

where $B:= (2C_r\bar{C}\root r \of {\vert Q\vert }M_{{\mathcal {U}}} + 1)$, $C_r$ is the constant of Lemma 1 and $\bar{C}$ is the one from Remark 4.

We proof an analogous statement for the adjoint state. For an elliptic state equation a similar result is proved in [3, Lemma 3.7].

### Lemma 31

Given ${\bar{u}} \in \mathcal {U}$ with associated state $y_{{\bar{u}}}$ and adjoint-state $p_{{\bar{u}}}$, there exists a positive constant ${\tilde{B}}$ such that

A7 $$\begin{aligned} \Vert p_{{\bar{u}} + \theta (u - {\bar{u}})} - p_{{\bar{u}}}\Vert _{L^\infty (Q)} \le {\tilde{B}}(\Vert y_u - y_{{\bar{u}}}\Vert _{L^\infty (Q)} +\vert m\vert \Vert u-{\bar{u}}\Vert _{L^1(Q)}^{\frac{1}{r}}), \end{aligned}$$

for all $$\theta \in [0,1] \text { and }u \in \mathcal {U}$$.

### Proof

Let us prove (A7). Given $u \in {\mathcal {U}}$ and $\theta \in [0,1]$, let us denote $u_\theta = {\bar{u}} + \theta (u - {\bar{u}})$, $y_\theta = y_{u_\theta }$, and $p_\theta = p_{u_\theta }$. Subtracting the equations satisfied by $p_\theta$ and $p_{{\bar{u}}}$ we get with the mean value theorem

$$\begin{aligned}&-\frac{d}{dt}(p_\theta - p_{{\bar{u}}})+{\mathcal {A}}^ *(p_\theta - p_{{\bar{u}}}) + \frac{\partial f}{\partial y}(x,t,{\bar{y}})(p_\theta - p_{{\bar{u}}}) \\&\quad = \frac{\partial L}{\partial y}(x,t,y_\theta ,u_\theta ) - \frac{\partial L}{\partial y}(x,t,y_{{\bar{u}}},{\bar{u}})+ \Big [\frac{\partial f}{\partial y}(x,t,y_{{\bar{u}}}) - \frac{\partial f}{\partial y}(x,t,y_\theta )\Big ]p_\theta \\&\quad = \Big [\frac{\partial ^2L}{\partial y^2}(x,t,y_\vartheta ) -p_\theta \frac{\partial ^2f}{\partial y^2}(x,t,y_\vartheta )\Big ](y_\theta - y_{{\bar{u}}})+m(u_\theta -{\bar{u}}), \end{aligned}$$

where $y_\vartheta = y_{{\bar{u}}}+ \vartheta (y_\theta - y_{{\bar{u}}})$ for some measurable function $\vartheta :Q \longrightarrow [0,1]$. Now, we can apply again Theorem 1 and Remark 4 to conclude from the above equation

$$\begin{aligned} \Vert p_\theta -p_{{\bar{u}}}\Vert _{L^\infty (Q)}&\le C_r(\bar{C} + M_{{\mathcal {U}}} \bar{C})\root r \of {\vert Q\vert }\Vert y_\theta - y_{{\bar{u}}}\Vert _{L^\infty (Q)}+\vert m\vert \theta C_r\Vert u-{\bar{u}}\Vert _{L^r(Q)}\\&\le {\tilde{B}}(\Vert y_u - y_{{\bar{u}}}\Vert _{L^\infty (Q)}+\vert m\vert \Vert u-{\bar{u}}\Vert _{L^1(Q)})^{\frac{1}{r}}, \end{aligned}$$

where ${\tilde{B}}:=C_r((\bar{C} + M_{{\mathcal {U}}} \bar{C})\vert Q\vert ^{\frac{1}{r}}B+(2M_{\mathcal {U}})^{\frac{r-1}{r}} )$, with *B* being the constant from Lemma 30. $\square$

### Proof of Lemma 10

The second variation of the objective functional is given by Theorem 6. Let us denote $u_\theta$, $y_\theta$, and $p_\theta$ as in the proof of Lemma 31. From (23) we obtain

$$\begin{aligned}&\vert [J''({\bar{u}} + \theta (u - {\bar{u}})) - J''({\bar{u}})](u - {\bar{u}})^2\vert \\&\quad \le \int _Q\Big \vert \Big [\frac{\partial ^2L_0}{\partial y^2}(x,t,y_\theta ) - \frac{\partial ^2L_0}{\partial y^2}(x,t,y_{{\bar{u}}})\Big ] z_{u_\theta ,u - {\bar{u}}}^2\Big \vert \,\textrm{d}x\,\textrm{d}t\\&\qquad + \int _Q\Big \vert (p_{{\bar{u}}}- p_\theta )\frac{\partial ^2f}{\partial y^2}(x,t,y_\theta )z_{u_\theta ,u - {\bar{u}}}^2\Big \vert \,\textrm{d}x\,\textrm{d}t\\&\qquad + \int _Q\Big \vert p_{{\bar{u}}}\Big [\frac{\partial ^2f}{\partial y^2}(x,t,y_{{\bar{u}}}) - \frac{\partial ^2f}{\partial y^2}(x,t,y_\theta )\Big ]z_{u_\theta ,u - {\bar{u}}}^2\Big \vert \,\textrm{d}x\,\textrm{d}t\\&\qquad + \int _Q\Big \vert \Big [\frac{\partial ^2L_0}{\partial y^2}(x,t,y_{{\bar{u}}})- p_{{\bar{u}}}\frac{\partial ^2f}{\partial y^2}(x,t,y_{{\bar{u}}})\Big ](z^2_{u_\theta ,u - {\bar{u}}} - z^2_{{\bar{u}},u - {\bar{u}}})\Big \vert \,\textrm{d}x\,\textrm{d}t\\&\qquad +2\Big \vert \int _Q (u-{\bar{u}})m\Big [ z_{u_\theta ,u - {\bar{u}}}-z_{{\bar{u}},u - {\bar{u}}}\Big ]\,\textrm{d}x\,\textrm{d}t\Big \vert \\&\quad = I_1 + I_2 + I_3 + I_4+I_5. \end{aligned}$$

Let us estimate the terms $I_i$, $i\in \{1,..,5\}$. For $I_1$, we deduce from Remark 4, (A6), (10) and (19) that for every $\rho _1 > 0$ there exists $\varepsilon _1 > 0$ such that

$$\begin{aligned} I_1 \le \rho _1\Vert z_{{\bar{u}},u - {\bar{u}}}\Vert ^2_{L^2(Q)}\quad \text {if}\quad \Vert y_u - y_{{\bar{u}}}\Vert _{L^\infty (Q)} < \varepsilon _1. \end{aligned}$$

We consider $I_2$. Let $m=0$, we use Remark 4, (10), (16), (19), and (A7) to obtain for every $\rho _2 > 0$ the existence of a $\varepsilon _2 > 0$ such that

$$\begin{aligned} I_2 \le \rho _2\Vert z_{{\bar{u}},u - {\bar{u}}}\Vert ^2_{L^2(Q)}\quad \text {if}\quad \Vert y_u - y_{{\bar{u}}}\Vert _{L^\infty (Q)} < \varepsilon _2. \end{aligned}$$

For the general case $m\in \mathbb {R}$, we use Remark 4, (10), (16), (19), and (A7), to infer for any $\rho _2>0$ the existence of a $\varepsilon _2>0$ such that

$$\begin{aligned} I_2&\le \bar{C}{\tilde{B}}(C_r(2M_{{\mathcal {U}}})^\frac{r-1}{r}+\vert m\vert )\Vert u-{\bar{u}}\Vert _{L^1(Q)}^{\frac{1}{r}} \Vert z_{{\bar{u}},u - {\bar{u}}}\Vert _{L^2(Q)}^2\\&\le \rho _2\Vert z_{{\bar{u}},u - {\bar{u}}}\Vert ^2_{L^2(Q)}\quad \text {if}\quad \Vert u - {\bar{u}}\Vert _{L^1(Q)} < \varepsilon _2. \end{aligned}$$

The estimate for $I_3$ follows from (10), (16), (19) and Remark 4. Thus for every $\rho _3>0$, there exists $\varepsilon _3>0$ with

$$\begin{aligned} I_3 \le \rho _3\Vert z_{{\bar{u}},u - {\bar{u}}}\Vert ^2_{L^2(Q)} \quad \text {if} \quad \Vert y_u - y_{{\bar{u}}}\Vert _{L^\infty (Q)} < \varepsilon _3. \end{aligned}$$

For $I_4$ we infer by Remark 4, (A3), (10), (16), (17), (19) and (A6) that for every $\rho _4 > 0$ there exists $\varepsilon _4> 0$ such that

$$\begin{aligned} I_4&\le (\bar{C} + M_{{\mathcal {U}}}\bar{C})\Vert z_{u_\theta ,u - {\bar{u}}} + z_{{\bar{u}},u - {\bar{u}}}\Vert _{L^2(Q)}\Vert z_{u_\theta ,u - {\bar{u}}} - z_{{\bar{u}},u - {\bar{u}}}\Vert _{L^2(Q)}\\&\le \frac{5C_{2}}{2}(\bar{C} + M_{{\mathcal {U}}}\bar{C})\Vert z_{{\bar{u}},u - {\bar{u}}}\Vert _{L^2(Q)}\Vert y_\theta -y_{{\bar{u}}}\Vert _{L^\infty (Q)}\Vert z_{{\bar{u}},u - {\bar{u}}}\Vert _{L^2(Q)}\\&\le \rho _4\Vert z_{{\bar{u}},u - {\bar{u}}}\Vert ^2_{L^2(Q)} \quad \text {if}\quad \Vert y_u - y_{{\bar{u}}}\Vert _{L^\infty (Q)} < \varepsilon _4. \end{aligned}$$

The term $I_5$ needs only to be considered in the general case $m\in \mathbb {R}$. We recall that in this case, we assume $\Vert u-{\bar{u}}\Vert _{L^1(Q)}$ to be sufficiently small. To estimate $I_5$ we use that $z_{{\bar{u}},v}$ satisfies equation (14) and that $\psi :=z_{{\bar{u}},u - {\bar{u}}}-z_{u_\theta ,u - {\bar{u}}}$ solves (A4). Then, by Remark 4, applying (10) to (A4), (16), (19), Lemma 2 and (A6) we estimate

$$\begin{aligned}&2 \Big \vert \int _Q (u-{\bar{u}})m\Big [ z_{u_\theta ,u - {\bar{u}}}-z_{{\bar{u}},u - {\bar{u}}}\Big ]\,\textrm{d}x\,\textrm{d}t\Big \vert \le 2\vert m\vert \Vert u - {\bar{u}}\Vert _{L^{s'}(Q)}\Vert z_{u_\theta ,u - {\bar{u}}}-z_{{\bar{u}},u - {\bar{u}}}\Vert _{L^s(Q)}\\&\quad \le 2\vert m\vert (2M_{{\mathcal {U}}})^{\frac{s'-1}{s'}} \Vert u - {\bar{u}}\Vert _{L^{1}(Q)}^{\frac{1}{s'}}\Vert z_{u_\theta ,u - {\bar{u}}}-z_{{\bar{u}},u - {\bar{u}}}\Vert _{L^s(Q)}\\&\quad \le 2\vert m\vert \bar{C}C_{s'}B(2M_{{\mathcal {U}}})^{\frac{s'-1}{s'}}\Vert u - {\bar{u}}\Vert _{L^{1}(Q)}^{\frac{1}{s'}} \Vert y_{u_\theta } - y_{{\bar{u}}}\Vert _{L^2(Q)}\Vert z_{{\bar{u}}_{\theta },u - {\bar{u}}}\Vert _{L^2(Q)}. \end{aligned}$$

We remark, that to make the last step, we used that (A6) holds also if the $\Vert \cdot \Vert _{L^\infty (Q)}$-norm is exchanged with the $\Vert \cdot \Vert _{L^2(Q)}$-norm. This can be seen in the proof of [3, Lemma 3.5]. Thus we infer that for every $\rho _5>0$ there exists a $\epsilon _5>0$ such that

$$\begin{aligned} I_5\le \rho _5 \Vert z_{{\bar{u}},u - {\bar{u}}}\Vert _{L^2(Q)}^2 \quad \text {if}\quad \Vert u - {\bar{u}}\Vert _{L^{1}(Q)} < \varepsilon _5. \end{aligned}$$

Now if $m=0$ the validity of the estimates for $I_i$ for $i\in \{1,...,4\}$ holds under the condition that $\Vert y_u - y_{{\bar{u}}}\Vert _{L^\infty (Q)}$ is sufficiently small. For general $m\in \mathbb {R}$ the validity of the estimates holds under the condition that $\Vert u-{\bar{u}}\Vert _{L^1(Q)}$ is sufficiently small by the additional arguments given above for the terms $I_2$ and $I_5$ and for the other terms by the fact that by (8), $\Vert u-{\bar{u}}\Vert _{L^1(Q)}<\frac{\varepsilon ^r}{C_r^r(2M_{\mathcal {U}})^\frac{r-1}{2r}}$, implies $\Vert y_u - y_{{\bar{u}}}\Vert _{L^\infty (Q)} < \varepsilon$. Taking $\varepsilon := \min _{1 \le i \le 5}\varepsilon _i$, completes the proof. $\square$

### Proof of Lemma 11

Let $s\in [1,\frac{n+2}{n})\cap [1,2]$. We first consider the case $m=0$. Using that $L_0$ and *f* satisfy the assumption in Remark 4 and arguing as in the proof of Lemma 10, there exists $\varepsilon >0$ and a positive constant *P* such that

$$\begin{aligned} \vert [J''({\bar{u}} + \theta (u - {\bar{u}})) - J''({\bar{u}})](u-\bar{u})^2\vert \le P \Vert y_u-y_{{\bar{u}}}\Vert _{L^\infty (Q)}\Vert z_{{\bar{u}},u-\bar{u}}\Vert _{L^2(Q)}^2 \end{aligned}$$

for all $u\in {\mathcal {U}}$ with $\Vert y_u-y_{{\bar{u}}}\Vert _{L^\infty (Q)}<\varepsilon$. To prove (40), we select $l_1,l_2\ge 0$ with $l_1+l_2=1$ and use the estimate

A8 $$\begin{aligned} \Vert z_{{\bar{u}},u-{\bar{u}}}\Vert _{L^2(Q)}\le \Vert z_{{\bar{u}},u-{\bar{u}}}\Vert _{L^\infty (Q)}^{\frac{2-s}{2}}\Vert u-{\bar{u}} \Vert _{L^1(Q)}^{\frac{s}{2}}. \end{aligned}$$

By (A8), (8), (10), (16) and (18) we find

$$\begin{aligned}&\Vert y_u - y_{{\bar{u}}}\Vert _{L^\infty (Q)} \Vert z_{{\bar{u}},u - {\bar{u}}}\Vert _{L^2(Q)}^2\\&\quad \le \Vert y_u - y_{{\bar{u}}}\Vert _{L^\infty (Q)} \Vert z_{{\bar{u}},u - {\bar{u}}}\Vert _{L^2(Q)} \Vert z_{{\bar{u}},u - {\bar{u}}}\Vert _{L^\infty (Q)}^{\frac{2-s}{2}}\Vert u-{\bar{u}}\Vert _{L^1(Q)}^{\frac{s}{2}}\\&\quad \le C_{s'}\sup _{{\mathcal {U}}} \Vert u - {\bar{u}}\Vert _{L^\infty (Q)}^{\frac{s-1}{s'}}\Vert y_u - y_{{\bar{u}}}\Vert _{L^\infty (Q)}^{l_1+l_2}\Vert z_{{\bar{u}},u - {\bar{u}}}\Vert _{L^2(Q)} \Vert u-{\bar{u}}\Vert _{L^1(Q)}^{\frac{2-s}{2s'}+\frac{s}{2}}\\&\quad \le C_{s'}^2{\tilde{M}}_{{\mathcal {U}}}\Vert y_u - y_{{\bar{u}}}\Vert _{L^\infty (Q)}^{l_1} \Vert z_{{\bar{u}},u - {\bar{u}}}\Vert _{L^2(Q)}\Vert u-{\bar{u}}\Vert _{L^1(Q)}^{\frac{l_2}{s'}+\frac{s}{2}}\Vert u-{\bar{u}}\Vert _{L^1(Q)}^{\frac{2-s}{2s'}}, \end{aligned}$$

with ${\tilde{M}}:=M_{{\mathcal {U}}}^{\frac{s-1}{s'}(l_2+\frac{2-s}{2})}$. We select $l_2$ such that

$$\begin{aligned} \frac{l_2}{s'}+\frac{2-s}{2s'}+\frac{s}{2}=1. \end{aligned}$$

We have that $1/s'=1-1/s$ is equivalent to $(1+l_2)(1-1/s)+s/2(1-1+1/s)=1$. Thus we find

$$\begin{aligned} l_2=s'/2-1. \end{aligned}$$

Defining $\varepsilon :=\frac{1}{C_{s'}^2{\tilde{M}}}\rho ^{\frac{1}{l_1}}$ proves the first claim. For the proof of (41) we use (A8), (8), (10), (16) and (18) to infer

A9 $$\begin{aligned} \begin{aligned}&\Vert y_u - y_{{\bar{u}}}\Vert _{L^\infty (Q)} \Vert z_{{\bar{u}},v}\Vert _{L^2(Q)}^2\le C_{s'} \Vert y_u - y_{{\bar{u}}}\Vert _{L^\infty (Q)}\Vert z_{{\bar{u}},v}\Vert _{L^\infty (Q)}^{(2-s)}\Vert u-{\bar{u}}\Vert _{L^1(Q)}^{s}\\&\quad \le C_{s'}^2M_{{\mathcal {U}}}^{\frac{s'-1}{s'}}\Vert y_u - y_{{\bar{u}}}\Vert _{L^\infty (Q)}^{l_1+l_2} \Vert u-{\bar{u}}\Vert _{L^1(Q)}^{\frac{2-s}{s'}}\Vert u-{\bar{u}}\Vert _{L^1(Q)}^{s}\\&\quad \le C_{s'}^3 {\tilde{M}}\Vert y_u - y_{{\bar{u}}}\Vert _{L^\infty (Q)} ^{l_1}\Vert u-{\bar{u}}\Vert _{L^1(Q)}^{\frac{l_2}{s'}} \Vert u-{\bar{u}}\Vert _{L^1(Q)}^{\frac{2-s}{s'}}\Vert u-{\bar{u}}\Vert _{L^1(Q)}^{s}, \end{aligned} \end{aligned}$$

with ${\tilde{M}}:=M_{{\mathcal {U}}}^{\frac{(s-1)(l_2+2-s)}{s'}}$. Select $l_2$ such that

$$\begin{aligned} \frac{l_2}{s'}+\frac{2-s}{s'}+s=2. \end{aligned}$$

By $1/s'=1-1/s$, this is equivalent to $l_2=(2-s)/(s-1)$. Setting $\varepsilon :=\frac{1}{C_{s'}^3{\tilde{M}}}\rho ^{\frac{1}{l_1}}$ proves the case for $m=0$. For $m\in \mathbb {R}$, we recall, that the $L^1(Q)$-distance of the controls is assumed to be sufficiently small. This is used to estimate the terms where the difference of the controls appears explicitly. For the terms where the controls do not appear explicitly we use the estimations for $m=0$ above and apply the estimate (8) to $y_u-y_{{\bar{u}}}$ to conclude that the $L^\infty (Q)$-distance of the states is close if the $L^1(Q)$-distance of the controls is close. $\square$

## Footnotes

1 For $p,r\in [1,\infty ]$, we speak of stability in the $L^p-L^r$-sense for the optimal states ${\bar{y}}$ with respect to perturbations (may appear in the equation or the objective) $\xi$, if there exists a positive constant $\kappa$ such that $\Vert y^{\xi }-{\bar{y}}\Vert _{L^p(Q)}\le \kappa \Vert \xi \Vert _{L^r(Q)}$, for all $\xi$ that are sufficiently small. Here, $y^\xi$ denotes the state corresponding to the perturbation $\xi$. We use this expression analogously for the optimal controls.
